# Temporal expression profiling of DAMPs-related genes revealed the biphasic post-ischemic inflammation in the experimental stroke model

**DOI:** 10.1186/s13041-020-00598-1

**Published:** 2020-04-07

**Authors:** Atsushi Yamaguchi, Tatsuya Jitsuishi, Takashi Hozumi, Jun Iwanami, Keiko Kitajo, Hiroo Yamaguchi, Yasutake Mori, Masaki Mogi, Setsu Sawai

**Affiliations:** 1 Department of Functional Anatomy, Graduate School of Medicine, Chiba University, 1-8-1 Inohana, Chuo-ku, Chiba, 260-8670 Japan; 2 Department of Orthopaedic Surgery, Graduate School of Medicine, Chiba University, 1-8-1 Inohana, Chuo-ku, Chiba, 260-8670 Japan; 3 Department of Molecular Cardiovascular Biology and Pharmacology, Ehime University, Graduate School of Medicine, 454 Shitsugawa, Toon, Ehime 791-0295 Japan; 4 Department of Neurology, Neurological Institute, Graduate School of Medical Sciences, Kyushu University, 3-1-1 Maidashi, Higashi-ku, Fukuoka, 812-8582 Japan; 5 International University of Health and Welfare School of Medicine, 4-3 Kozunomori, Narita, Chiba, 286-8686 Japan; 6 Department of Pharmacology, Ehime University, Graduate School of Medicine, 454 Shitsugawa, Toon, Ehime 791-0295 Japan

**Keywords:** Ischemic stroke, DAMPs, Sterile neuroinflammation

## Abstract

The neuroinflammation in the ischemic brain could occur as sterile inflammation in response to damage-associated molecular patterns (DAMPs). However, its long-term dynamic transcriptional changes remain poorly understood. It is also unknown whether this neuroinflammation contributes to the recovery or just deteriorates the outcome. The purpose of this study is to characterize the temporal transcriptional changes in the post-stroke brain focusing on DAMPs-related genes by RNA-sequencing during the period of 28 days. We conducted the RNA-sequencing on day 1, 3, 7, 14, 28 post-stroke in the mouse photothrombosis model. The gross morphological observation showed the ischemic lesion on the ipsilateral cortex turned into a scar with the clearance of cellular debris by day 28. The transcriptome analyses indicated that post-stroke period of 28 days was classified into four categories (I Baseline, II Acute, III Sub-acute-#1, IV Sub-acute-#2 phase). During this period, the well-known genes for DAMPs, receptors, downstream cascades, pro-inflammatory cytokines, and phagocytosis were transcriptionally increased. The gene ontology (GO) analysis of biological process indicated that differentially expressed genes (DEGs) are genetically programmed to achieve immune and inflammatory pathways. Interestingly, we found the biphasic induction of various genes, including DAMPs and pro-inflammatory factors, peaking at acute and sub-acute phases. At the sub-acute phase, we also observed the induction of genes for phagocytosis as well as regulatory and growth factors. Further, we found the activation of CREB (cAMP-response element binding protein), one of the key players for neuronal plasticity, in peri-ischemic neurons by immunohistochemistry at this phase. Taken together, these findings raise the possibility the recurrent inflammation occurs at the sub-acute phase in the post-stroke brain, which could be involved in the debris clearance as well as neural reorganization.

## Introduction

Post-stroke neuroinflammation occurs in the absence of invading pathogens. Damage-associated molecular patterns (DAMPs) are considered as sources for the sterile inflammation in the post-ischemic brain [1–3]. However, the long-term dynamic temporal expression changes of DAMPs-related genes are poorly understood. It is also unknown whether this sterile neuroinflammation contributes to the recovery or just deteriorates the outcome.

The cellular response in the neuroinflammation is linked to the glial activation [4]. Inflammation-mediated neurotoxicity is considered to occur as consequences of the glial dysregulation and over-activation in response to DAMPs, while moderate central nervous system (CNS) damages could confer protection [1]. Cytokines are key modulators of inflammation, participating in acute and chronic inflammation via a complex network of interactions. Key pro-inflammatory cytokines include interleukin-1 (IL-1), IL-6, and tumor necrosis factor (TNFα), while anti-inflammatory mediators contain IL-10 and TGF-β [5].

DAMPs comprise a quite diverse group of dis-compartmentalized self-structures and ECM (extracellular matrix) [6, 7]. Pattern recognition receptors (PRRs), initially discovered for their role in recognizing PAMPs (pathogen-associated molecular patterns), relate to the sterile inflammation in response to DAMPs. They consist of diverse family members, including Toll-like receptors (TLRs), Nod-like receptors (NLRs), RIG-like receptors (RLRs), AIM2-like receptors (ALRs), C-type lectin receptors (CLECs), and the receptor for advanced glycation end products (RAGE). TLR signaling, for instance, is responsible for the induction of pro-inflammatory response thorough the downstream pathways, such as inflammasome (e.g., PYCARD), interferon regulatory factors (IRFs), and TRAFs [8, 9].

As one of immune defense in the CNS, the phagocytosis by microglia is extensively studied [10]. In the brain as an immune-privileged space, the detection and clearance of cellular debris in ischemic insult are owed to the innate immune system. The activity of microglial phagocytosis relies on specific receptors on the cell surface, including TLRs, CD36 (scavenger receptor class B member 3), triggering receptor expressed on myeloid cells 2 (TREM-2), Fc receptors, complement receptors, scavenger receptors (SR), and mannose receptor. Enhanced phagocytic activity leads to reduce the expression of pro-inflammatory mediators and increase the production of anti-inflammatory mediators, resolving the neuroinflammation [10, 11]. Thus, despite the role of establishing neuroinflammation, glial cells harbor a protective role to maintain homeostasis and resolve neuroinflammation [12, 13].

In the present study, we profiled the long-term temporal gene expression in post-ischemic brains focusing on DAMPs-related genes in the mouse stroke model. We then found the biphasic induction of inflammatory response peaking at the acute and sub-acute phases in the post-ischemic brain.

## Material and methods

### Photothrombosis stroke model

Photothrombosis was performed in male 6–8 weeks old C57/BL6J mice (20–22 g) from Japan SLC Inc. as described previously [14]. Briefly, mice were deeply anesthetized, and Rose Bengal (1 mg) (TCI chemicals, Japan) was injected intraperitoneally at 5 min before illumination. Mice were placed in a stereotactic frame (NARISHIGE #SR-5 M, Japan), and the skull was exposed by a median incision of the skin to identify bregma and lambda points. A fiber optic bundle of a cold light source (Zeiss Cold light source CL 1500) with × 20 objective lens (Olympus), centered using a manipulator at 2 mm laterally from bregma (sensorimotor cortex), illuminated the brain through the intact skull for 15 min initiating at 5 min after the injection of Rose Bengal. The body temperature was controlled during the operation by a heating pad. Sham operations were performed in parallel without illumination by a cold light source. Sham-operated mice at day 7 post-operation were used as control. All efforts were made to minimize animal suffering and the number of animals used (*n* = 3–5 /group at each time point) in photothrombosis stroke model. Experiments with animals were approved by the institutional Animal Care and Use Committee at Chiba University.

### Immunohistochemistry

Mice were intracardially perfused with 4% paraformaldehyde (PFA). Brains were harvested and post-fixed in 4% PFA overnight at 4 °C, followed by sucrose replacement. Brain sections, cut in 20 μm-thick sections with a cryostat, were permeabilized in PBS (Phosphate Buffered Saline) containing 0.2% Triton-X 100 with 5% bovine serum albumin (BSA) with non-specific sites blocked in blocking solution [PBS containing 0.1% Triton X-100, 5% BSA]. The samples were incubated with a primary antibody diluted in a blocking solution overnight at 4 °C. The primary antibodies used were anti-GFAP (Sigma-Aldrich, # G9269), anti-Iba1 (WAKO, # 019–19,741), anti-pCREB (Ser133) (87G3) (Cell Signaling Technology, # 9198), anti-CREB (Cell Signaling Technology, # 9197), Anti-GAP43 (phospho Ser41) (Bioss Inc., #bs-1641R), and anti-GAP43 (Invitrogen, PA5–79299). After washed in PBS, sections were treated with Vectastain ABC Elite kit (Vector Laboratories, Burlingame, CA, USA) according to manufacturer’s instructions. After the last three washes, sections were developed using DAB (3, 3-diaminobenzidine) substrate solution. Images were obtained using fluorescence microscopy (Nikon, E600) with digital camera DP72 (Olympus, Tokyo, Japan). Photographs of the DAB-stained brain sections were analyzed by Image J software (http://rsbweb.nih.gov/ij/download.html) to semi-quantify the anti-pCREB antibody-positive areas. The four independent areas were compared for their intensities of pCREB-positive area/analyzed area, and data were expressed as fold change vs. contra-lateral cortex ± SEM.

### Cresyl violet staining (Nissl staining)

The brain section on the slide was immersed through 100% ethanol for 3 min with two changes. To defat the tissue, slide were processed in 100% xylene for 15 min and then in 100% ethanol for 10 min. Then the slide was dehydrated through 100% alcohol for 3 min twice, washed in tap water, and stained in 0.1% Cresyl Violet (Muto Pure Chemicals, Tokyo) for 4–15 min at 37 °C. After a quick rinse in tap water to remove excess stain, the slide was washed in 70% ethanol (the stain was removed by this method), dehydrated through a standard series of ethanol, cleared in xylene twice, and mounted on slide glass for microscopic observation.

### Western blot analysis

Western blot analysis was performed as described previously [14]. Brain lysates for western blot analysis were prepared by lysis buffer [50 mM Tris-HCl (pH 8.0), 20 mM EDTA, 1% NP-40, 100 mM NaCl, 10 mM β*-*glycerophosphate*,* Complete Protease Inhibitor Cocktail (Roche Diagnostics)]. The samples (30 μg/lane) were boiled in loading buffer [100 mM Tris-HCl (pH 6.8), 200 mM dithiothreitol, 4% SDS, 0.2% bromophenol blue, 0.2% glycerol] for 5 min, and subjected to electrophoresis on 10% SDS-PAGE. After the proteins were transferred onto polyvinylidene difluoride (PVDF) membrane (Millipore Corp.), the membrane was incubated in blocking buffer [phosphate-buffered saline (PBS) containing 0.05% Tween 20 (PBS-T) with 5% nonfat dried milk] for 1 h at room temperature and then probed with a primary antibody in blocking buffer overnight at 4 °C. The membrane was washed three times in PBS-T, probed with the secondary horseradish peroxidase-linked anti-mouse or -rabbit IgG antibody (Cell Signaling Biotechnologies) in blocking buffer for 1 h at room temperature, and washed again in PBS-T. Detection of signal was performed with ECL chemiluminescence system (Thermo Fisher Scientific). The intensities of bands were analyzed by Image J software for semi-quantification. Data were expressed as fold change vs control (sham-operated mice) ± SEM, and *p* values were determined with Student’s t-test (*p < 0.05 was considered significant).

### RNA isolation

Total RNAs were extracted from the whole ipsilateral cortex with RNAiso Plus (Takara Bio., Japan) and cleaned with NucleoSpin RNA purification kit (Takara Bio., Japan) with on-column DNase I digestion. RNA Integrity Numbers (RIN) was assessed using Agilent 2100 Bioanalyzer (Agilent Technologies, Palo Alto, CA). This indicator was > 8 in all the samples.

### RNA-sequencing library construction, sequencing, and analyses

The total RNAs of 3 mice were pooled together at each time, including control (sham-operated mice). RNA samples were sent to the GENE WIZ (Saitama, Japan) for RNA-seq analyses. Experimental procedures of RNA-seq are available in the Supplementary data. A detailed list of the DEGs detected in each of the comparison was presented in Supplementary Tables S2, S3, S4, S5 and S6.

### Data availability

The raw RNA-seq data have been deposited in NCBI’s Gene Expression Omnibus (GEO) and are accessible through GEO Series accession number GSE147060.

### Real-time PCR assay

Total RNAs were extracted from the whole ipsilateral cortex of sham-operated (control) or photothrombosis (PT) mice at day 1, 3, 7, 14, 28 post-stroke using RNAIso-PLUS (Takara Bio, Japan). RNA (0.5 μg) was reverse transcribed to produce cDNA using ReverTra Ace® qPCR RT Kit with gDNA remover (TOYOBO, Japan). Real-time PCR was performed using THUNDERBIRD® SYBR® qPCR Mix (TOYOBO, Japan). All real-time PCR assays were performed in biological triplicate using 7300 Real-Time PCR System (Applied Biosystems). Primers in the study were listed on Supplementary Table S1. For standardization of relative mRNA expression, 18 s ribosomal primers were used. The results of cycle threshold values (Ct values) were calculated by the ΔΔCt method to obtain the fold differences. Five brains were used for real-time PCR analysis at each time point. Data were expressed as fold changes vs. control (sham-operated mice) ± SEM. The asterisks indicate a statistically significant difference from the control value (*p < 0.05, **p < 0.01 vs. control, Dunnett’s multiple comparison test of Biocunductor in R studio).

## Results

### Gross morphology and glial activation in the post-stroke brain

**Fig. 1:**
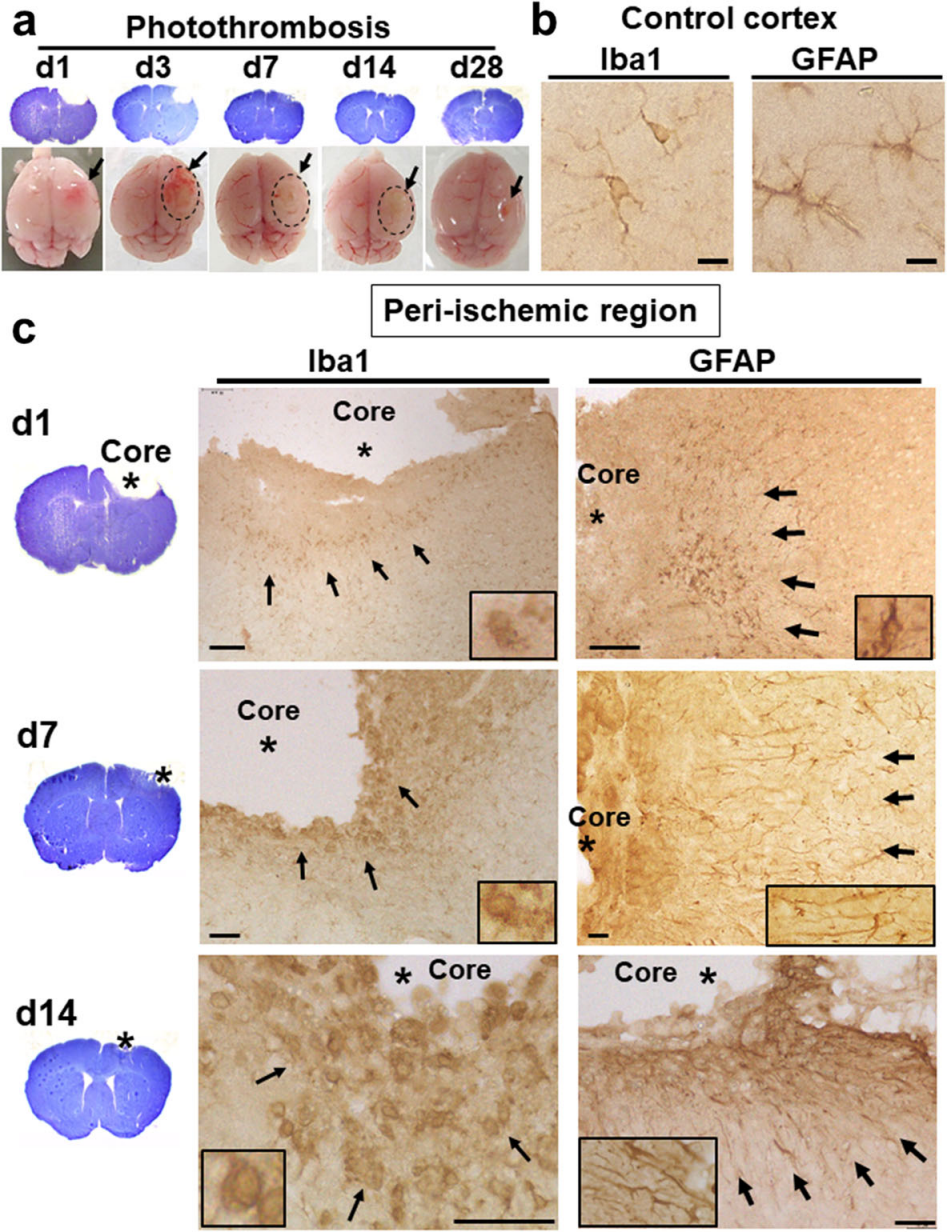
Gross morphology and glial activation on brain sections of the post-stroke brain. **a** Cresyl violet-stained brain sections and mouse brains at day 1, 3, 7, 14, and 28 post-stroke. Arrows indicate the ischemic regions. Dotted circles show the debris covering the ischemic lesion. **b** Typical ramified microglia and resting astroglia on the control (sham-operated) cortex. Immunostaining with anti-Iba1 and anti-GFAP antibody on the control cortex, respectively. Scale bar = 10 μm. **c** Activation of microglia and astroglia in the post-stroke brain. Immunostaining with anti-Iba1 and anti-GFAP antibody in the peri-ischemic region of the ipsilateral cortex at day 1, 7, and 14 (d1, d7, d14), respectively. Scale bar = 10 μm (middle panels), 100 μm (upper and lower panels). The higher magnification images are shown in the rectangles

The rodent photothrombosis stroke model is widely used to study the pathophysiology of ischemic stroke [14]. We first examined the temporal morphological changes in post-ischemic brains for 28 days after photothombosis (Fig. 1a). The ipsilateral cerebral cortices tended to swell possibly due to the brain edema from day 1 to 7 post-stroke (Fig. 1a), which were semi-quantified gross morphologically in Supplementary Fig. S6a, b. The large round debris covered the ischemic lesion from day 3 to 14, and then ischemic lesion turned into a scar by day 28 (Fig. 1a, lower panels and Supplementary Fig. S6a). The Nissl (cresyl violet) staining could define the neuronal loss and disorganization of the infarct region [15]. The Nissl-stained coronal sections showed the infarct areas, which were artificially defected during the preparation and decreased gradually with the reduction of brain swelling (Fig. 1a, upper panels).

Reactive gliosis is a hallmark of brain pathology in response to ischemic insult, which specifically refers to progressive changes in the gene expression and cellular morphology of astroglia and microglia [16]. To examine the immune response, we performed the immunohistochemistry with antibodies for glial markers, including anti-acidic fibrillary protein (GFAP) antibody for astrocytic activation and anti-ionized calcium-binding adapter molecule (Iba1) antibody for microglial activation (Fig. 1b, c). Astroglial cells, consisting of various subtypes (e.g., protoplasmic and fibrous type), extend long and ramified processes in resting state [17]. Resting microglia also harbors long, thin, and highly ramified processes. We observed typical resting astroglia and microglia on the cerebral cortex of the sham-operated (control) mouse (Fig. 1b).

As early as day 1 post-stroke, the dense immune-reactivity with anti-Iba1 as well as anti-GFAP antibody was detected around the ischemic core (Fig. 1c, upper panels). Since microglial activation precedes and predominates over macrophage infiltration in rodent stroke model [18], Iba1-positive cells, although including blood-derived monocytes/macrophages, were considered to be mainly microglial cells. Activated microglia, more rounded, hypertrophic, and amoeboid-like structure, appeared in peri-ischemic regions (Fig. 1c, left panels). The number of Iba1-positive cells was increased in the peri-ischemic regions as compared to the contralateral side peaking at day 7 post-stroke (Supplementary Fig. S6c, d). Glial scar, where the infarct core is surrounded by reactive astroglia, appeared by day 7 (Fig. 1c, right panels). At day 7 and 14, astroglial cells in peri-ischemic region showed long fibrillary shape extending toward the ischemic core (Fig. 1c, right panels), consistent with previous reports [19, 20].

### Temporal gene expression profiling in the post-stroke brain

**Fig. 2:**
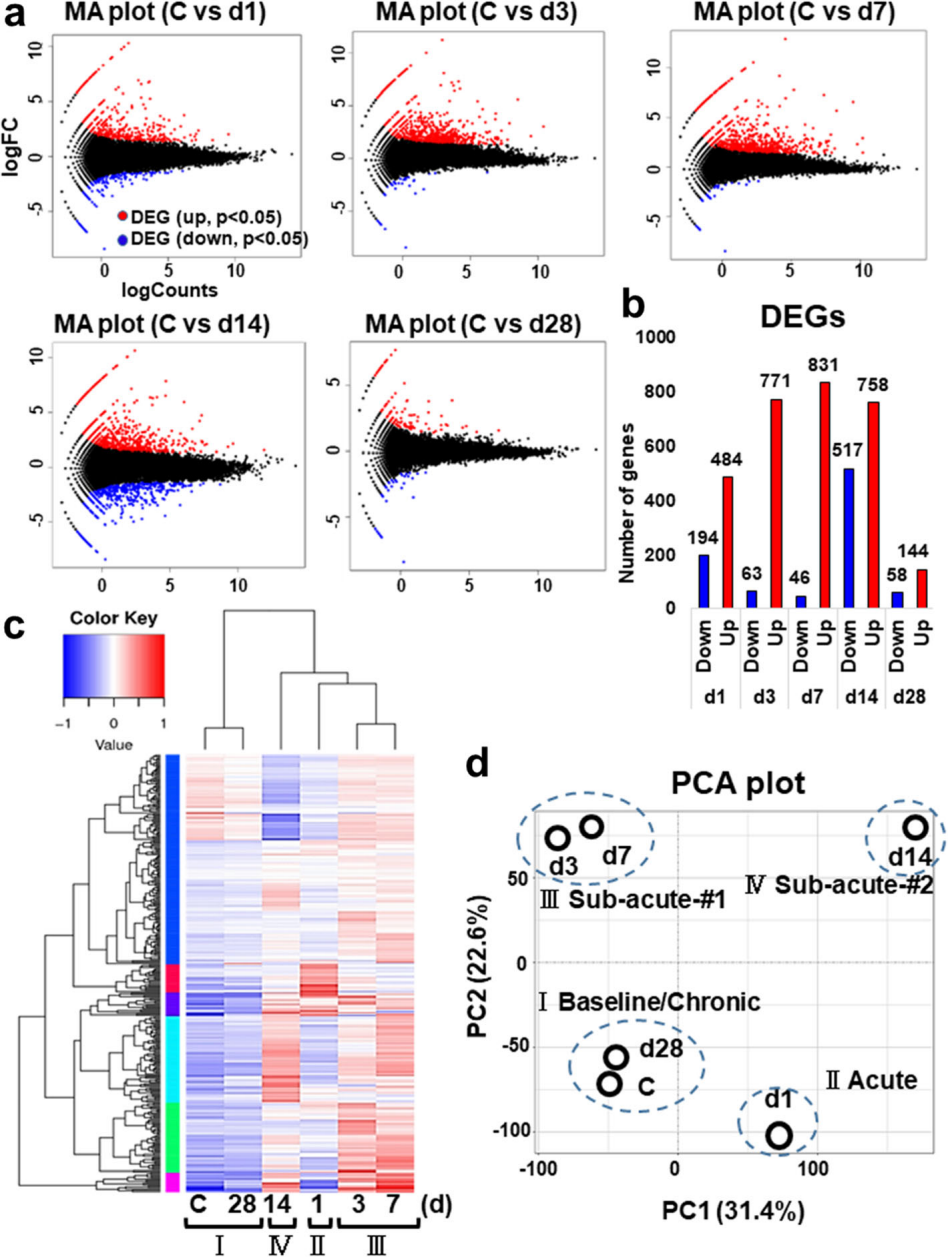
Transcriptome analyses in the post-stroke brain. **a** The MA plots indicate the differentially expressed genes (DEGs) in each comparison with control (C; sham) at day 1, 3, 7, 14, and 28 post-stroke (d1, d3, d7, d14, d28), respectively. DEG; differentially expressed gene. Up- (red) and down-regulated (blue) genes are dotted in each color. **b** The histogram shows the differentially expressed genes (DEGs) that were up- or down-regulated at each time point, respectively. **c** The heatmap shows the level of gene expression [in log10 (FPKM+ 1)] across the 6 temporal samples (C, d1, d3, d7, d14, d28) and categorizes them into 4 groups dependent on the gene expressing patterns in the dendrogram. Up- and down-regulated genes are colored in red and blue, respectively. The regions of different colors represent different clusters. FPKM, Fragments Per Kilobase of transcript per Million mapped reads. Control (C); sham-operated mice. **d** Primary component analysis (PCA) plot clustered 6 temporal samples (C, d1, d3, d7, d14, d28) into 4 categories (I ~ IV) dependent on principal component 1 (PC1, X-axis, 31.4%) and 2 (PC2, Y-axis, 22.6%)

Next, to investigate the immune response to ischemic insult transcriptionally, we then performed RNA-seq analyses at day 1, 3, 7, 14, and 28 post-stroke. The pairwise comparison of control (sham operation) versus stroke sample was conducted at each time point (Fig. 2a). The MA plot highlights genes that were differentially expressed in each comparison by plotting averaged expression (log Counts) against fold-change (log FC) for the individual gene at each time point (Fig. 2a). The numbers of differentially expressed genes (DEGs) at each time point were represented in the bar graph (Fig. 2b). The full list of DEGs in each pairwise comparison was shown in Supplementary Table S2, S3, S4 and S5. The ipsilateral cortices differentially expressed 678 (up 484, down 194), 834 (up 771, down 63), 877 (up 831, down 46), 1275 (up 758, down 517), and 201 (up 144, down 58) genes compared to the control (*p* < 0.05) at day 1, 3, 7, 14, and 28, respectively. On the other hand, the number of down-regulated genes was the largest at day 14 post-stroke.

The heat map provides a visual representation of expression levels for each gene. The hierarchical clustering classified the 6 temporal samples (Control, day 1, 3, 7, 14, 28) into the 4 categories (I ~ IV) (Fig. 2c). The principal component analysis (PCA), in which the first two principal components account for a total of 54%, also revealed the 6 temporal samples are segregated into 4 categories (I Baseline/Chronic, II Acute, III Sub-acute-#1, IV Sub-acute-#2) dependent on principal component 1 (PC1, X-axis, 31.4%) and 2 (PC2, Y-axis, 22.6%) (Fig. 2d). The transcriptomic data at day 28 harbor a similar tendency to those of control, which is consistent with the gross morphological observation the ischemic lesion apparently turned into a scar by day 28 post-stroke.

### Top up-regulated genes and GO biological processes in the post-stroke brain

**Fig. 3:**
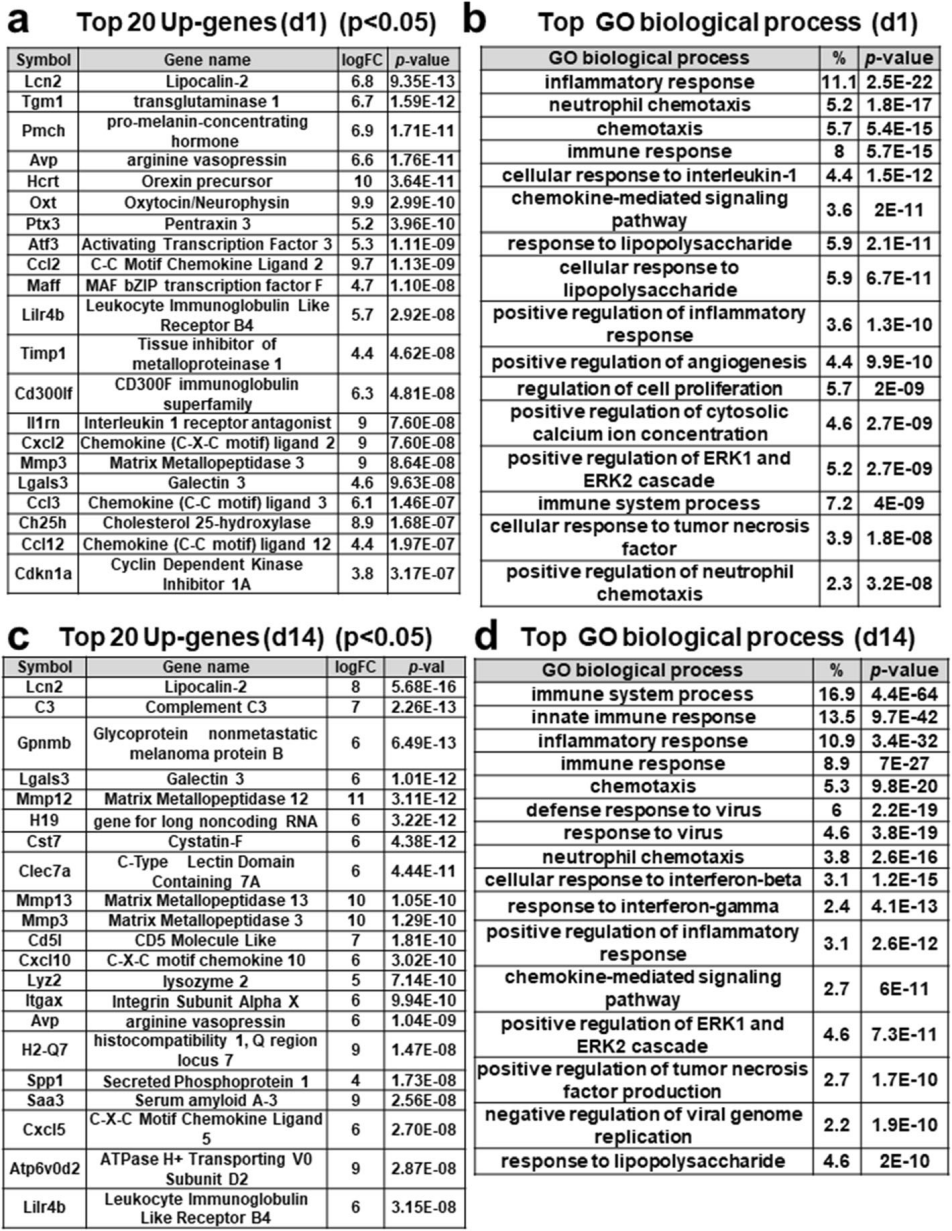
Top up-regulated genes and GO biological processes at day 1 and 14 (d1, d14) post-stroke. **a**, **c** Table showing top-upregulated genes (*p* < 0.05) at day 1 and 14 post-stroke compared with control (sham-operated mice), respectively. *logFC means log2 (fold changes). **b**, **d** Table showing top-upregulated GO biological processes (*p* < 0.05) enriched at day 1 and 14 post-stroke, which was identified by DAVID, respectively. *(%) means the ratio of up-regulated genes in each biological process per total genes

The lists of top differentially expressed genes (DEGs) compared with control (*p* < 0.05) at the acute (day1) and sub-acute phases (day 14) are shown in Fig. 3a and c. Those lists at day 3 and 7 are in Supplementary Fig. S1. The representative top up-regulated genes, at the acute and sub-acute phases, were those, encoding Lipocalin-2 (*Lcn-2*), cytokines (*Ccl2, Ccl3, Ccl12, Cxcl2, Cxcl5*), metalloproteases (*Mmp3, 12, 13*), toll-like receptor (*Tlr8*), c-type lectin domain containing protein (*Clec7a*), transgluminase1 (*Tgm1*), Complement (*C3*), lysosomal enzyme (*Lyz2*), galectin family (*Lgals3*), cholesterol 25-hydroxylase (*Ch25h*), hormones (*Oxt, Avp*), phagocytosis-related receptor (*Msr1; Macrophage Scavenger Receptor1*), and putative tissue repair-related genes (*Gpnmb*, *Spp1*) (Fig. 3a, c). The genes for regulatory factors of inflammation were also induced at day 7–14, including *Cd5l* and Long Non-coding RNA *H19* [21, 22] (Fig. 3c, Supplementary Fig. S1). Gene Ontology (GO) biological processes, significantly enriched at the acute and sub-acute phases, were a variety of immune- and inflammatory-associated processes (Fig. 3b, d and Supplementary Fig. S1). On the other hand, the number of down-regulated genes was the largest at day 14 (Fig. 2b) with significant enrichment in biological processes, including adhesion molecules, potassium ion transport, regulation membrane potential, chemical synaptic transmission, and neuron migration (Supplementary Fig. S5).

To study the corresponding changes of neuroinflammation in this stroke model, we examined the temporal expression changes of neuroinflammatory response-related genes in MGI Gene Ontology Browser (http://www.informatics.jax.org/vocab/gene_ontology). The neuroinflammatory response is defined as “the immediate defensive reaction by neural tissue to infection or injury caused by chemical or physical agents (ID GO:0150076)”. The 69 genes are classified as neuroinflammatory response-related genes in the GO functional annotation (ID GO:0150076), which are categorized into glial cell activation, negative or positive regulation, and reactive gliosis. Some of these genes overlap with the DAMPs-related genes in our study. Then we investigated the temporal expression profile of these 69 genes in the data of our RNA-seq and summarized the results in Supplementary Fig. S7. The neuroinflammatory response-related genes were induced peaking at day 1 to 14 post-stroke, including microglial cell activation (*Tlr1–8*, *Tnf, Tyrobp*), positive regulation of microglial cell activation (*Mmp8*), negative regulation (*Cst7*) and regulation (*cd200r2, cd200r3, cd200r4*) of neuroinflammatory response, and astrocyte activation (*C1qa, C5ar1, Fpr2, Grn, Il1b, and Trem2*) (Supplementary Fig. S7).

### DAMPs as a possible source for the sterile inflammation

**Fig. 4:**
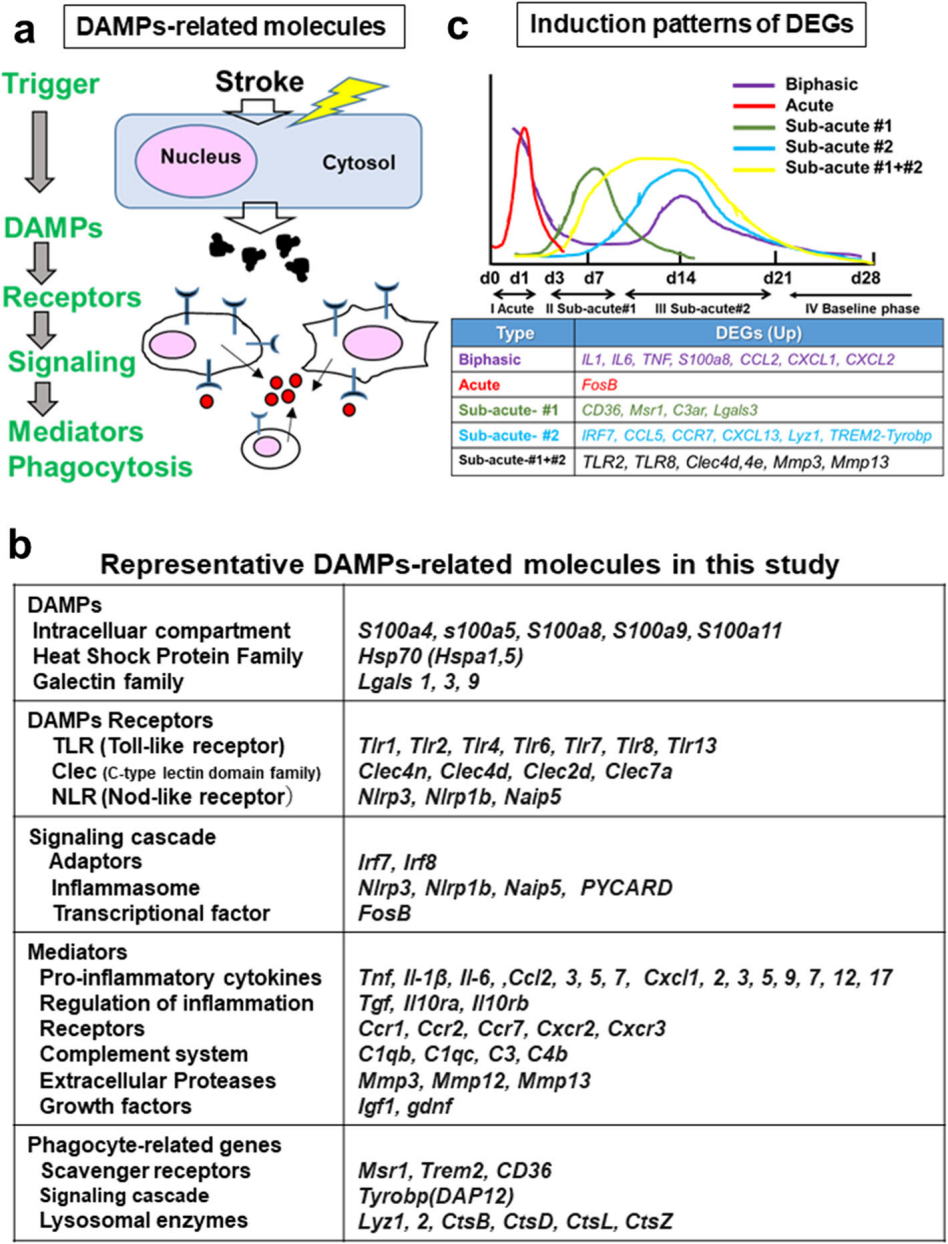
DAMPs-related molecules in the stroke brain. **a** The schematic diagram indicates the DAMPs-related molecules in the stroke brain. **b** The table showing DAMPs-related molecules that were up-regulated (*p* < 0.05, RNA-seq) in the present study. **c** The graphs categorized DEGs into 5 groups dependent on the expression patterns, including Biphasic, Acute, Sub-acute#1, Sub-acute#2, and Sub-acute#1 + #2 pattern

**Fig. 5:**
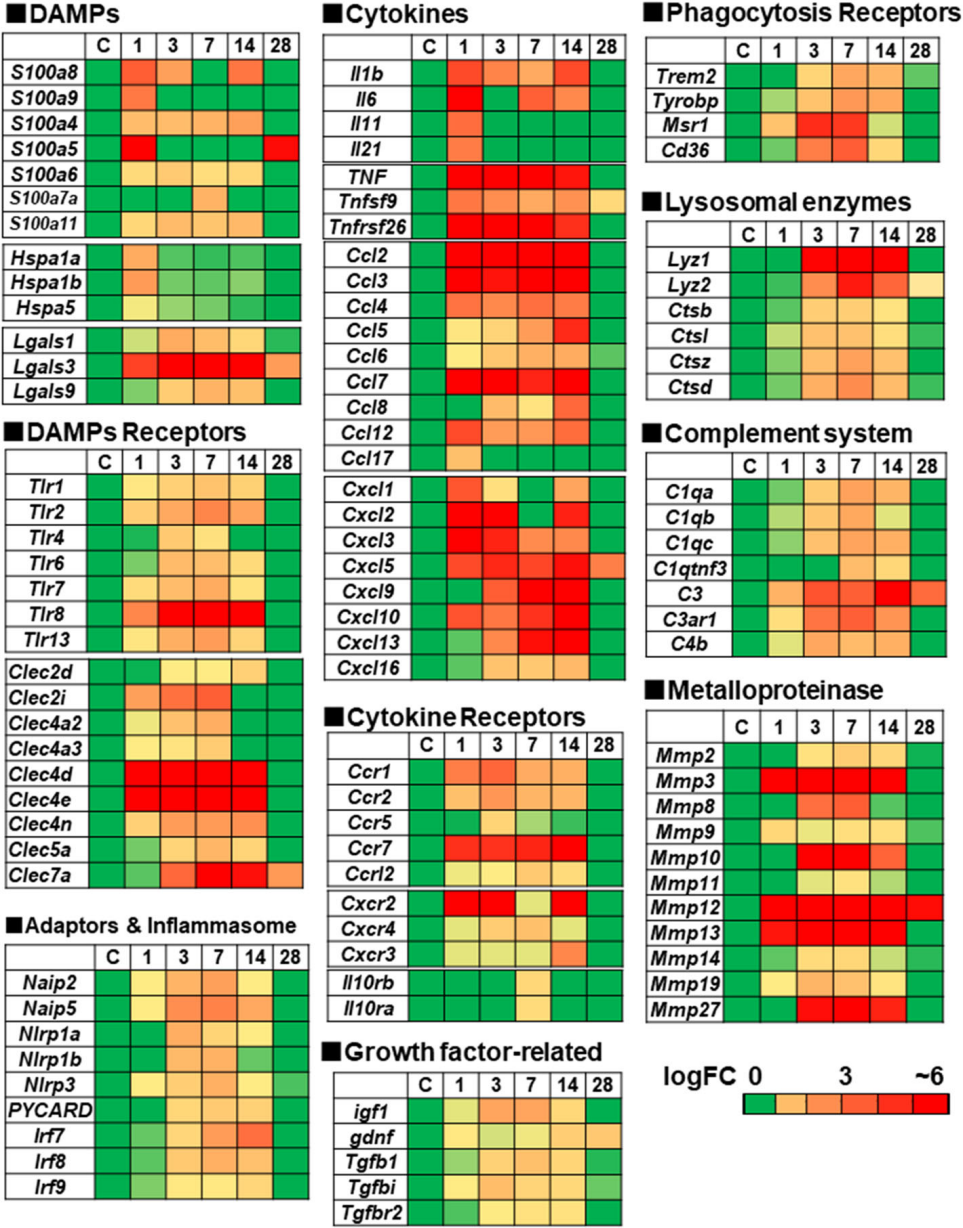
Heatmaps showing the up-regulated genes for representative DAMPs-related molecules in the post-stroke brain. The temporal profiles for genes that are transcriptionally induced (*p* < 0.05) were categorized in each heatmap (gene name in row, logFC in column at each time point of control and day 1, 3, 7, 14, 28 post-stroke). The coloring range indicates the value of logFC. logFC means log2 (fold changes)

DAMPs comprise a quite diverse group of discompartmentalized self-structures and ECM [6, 7]. We here classified DAMPs-related genes into several categories depending on the hierarchical cascades, including DAMPs, receptors, downstream cascades, mediators, and phagocytosis as shown in the schematic diagram (Fig. 4a). The table in Fig. 4b shows representative DAMPs-related genes that were induced in this study (*p < 0.05* in RNA-seq). The heat maps in Fig. 5 indicate the temporal expression profiles of the DAMPs-related genes, while the schematic diagram in Fig. 4c represents the induction patterns of DEGs in the present study (RNA-seq and qRT-PCR).

#### DAMPs

Well-known members of *DAMPs*, including *S100 family* (e.g., *S100a4, 8, 9*) were induced, peaking at day 1 and/or 14 post-stroke (Fig. 5). *Lgals3*, encoding Galectin-3 that can activate TLR4/NF-κB signaling, was induced peaking at day 3–14 [23].

#### Receptor (PRRs)

*Clec4d* and *Clec4e* were up-regulated at day 1–14, while *Tlr 2, 4, 6, 7, 8, 13* and *Clec7a* were induced peaking later after day 3 (Fig. 5).

#### Adaptors and inflammasome

PRRs interact with specific adaptors or “inflammasome” for transmitting the signals. *Irf7*, *Irf8* (adaptors) and *Nlrp3*, *Naip5*, *PYCARD* (inflammasome) were induced mainly at day 3–14 (Fig. 5).

#### Mediators

**Fig. 6:**
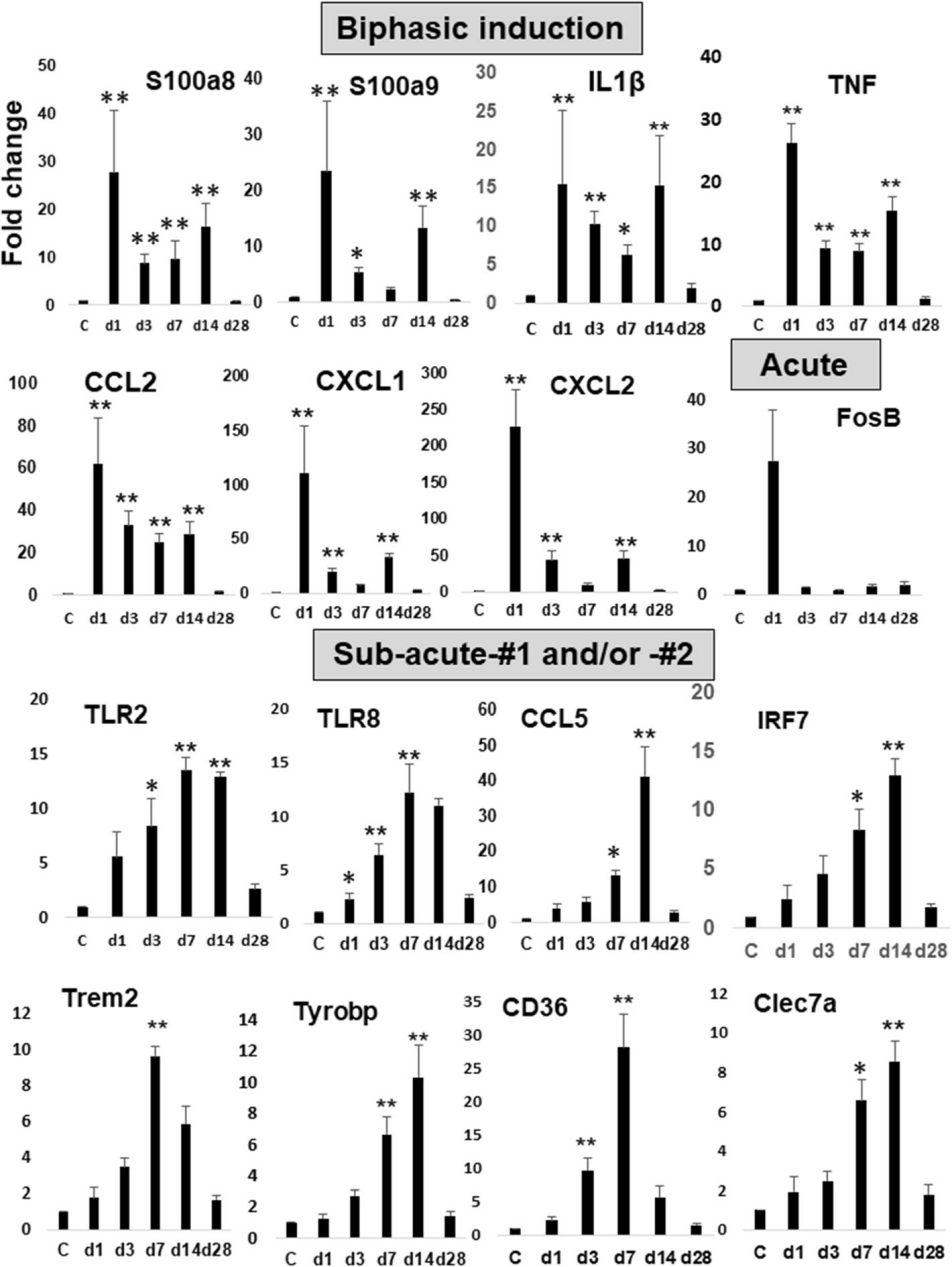
Histograms of real-time RT-PCR results for the representative DAMPs-related genes. Histograms show the results of qRT-PCR at each time point (day 1, 3, 7, 14, 28 post-stroke). The results of cycle threshold values (Ct values) were calculated by the ΔΔCt method to obtain the fold differences. Five brains derived from each group (control and photothrombosis) were used for real-time PCR analysis at each time point. Data are expressed as fold change relative to sham-operated mice (control). The bars represent the mean ± SEM. The asterisks indicate a statistically significant difference compared with control (*p < 0.05, **p < 0.01 versus control, Dunnett’s multiple comparison test)

Cytokines are key modulators of inflammation. A variety of genes for chemokines and their receptors (*Ccl*, *CxCl*, *Ccr*, and *Cxcr* family) were induced acutely or sub-acutely (Fig. 5). The genes (*Il-1, Il-6, tnf*), encoding critical pro-inflammatory cytokines, were induced from the acute phase, while those encoding regulatory molecules (*Ifnβ, Tgfβ*) were induced sub-acutely at day 3–14 (Fig. 5 and Supplementary Fig. S3). *FosB*, encoding subunits of the activator protein-1 transcription factor complex, is involved in the excitotoxic activation in microglia [24]. We found the acute induction of *FosB* (shown later in Fig. 6). We also observed the up-regulation of *metalloproteases* (*Mmp2, 3, 8, 10, 12, 13, 27*) at day 3–14.

#### Complement

Complement (C) is a major component of innate immunity, recognizing danger, as well as discriminating self from non-self [25]. The complement peptide C3a stimulates neural plasticity after experimental brain ischemia [26]. The genes, encoding C1qa, C1qb, C1qc, C3, C3ar1, and C4b, were induced peaking at day 3–14 (Fig. 5).

#### Phagocytosis

Phagocytosis is a receptor and ligand-mediated process. We found the induction of genes for scavenge receptors, including *Trem-2*, *Msr1* (macrophage scavenger receptor 1), and *Cd36* (scavenger receptor class B member 3) (Fig. 5). Genes for lysosomal enzyme (*Lyz1, Lyz2, Ctsb, Ctsl, Ctsz, Ctsd*) (Fig. 5) and H^+^ pump (*Atp6v 0d2*) (Fig. 3c) were induced peaking at day 3–14.

#### Growth factors

We found the induction of *Insulin-like growth factor* (*igf1*), and *Glial Cell Line-derived Neurotrophic Factor* (*gdnf*) (Fig. 5).

### The validation by quantitative real-time PCR assay

To validate the results of RNA-seq analyses, we performed the quantitative real-time PCR (qRT-PCR) on a subset of DEGs. We found the significant induction of approximately ~ 75% of DEGs at some point by qRT-PCR assay (Supplementary Figs. S2, S3, S4). We showed representative results of qRT-PCR in Fig. 6 after categorizing them into the three groups (Biphasic, Acute, Sub-acute-#1 and/or -#2) depending on the induction patterns.

### The activation of CREB and GAP43 in the ipsilateral cortex

**Fig. 7:**
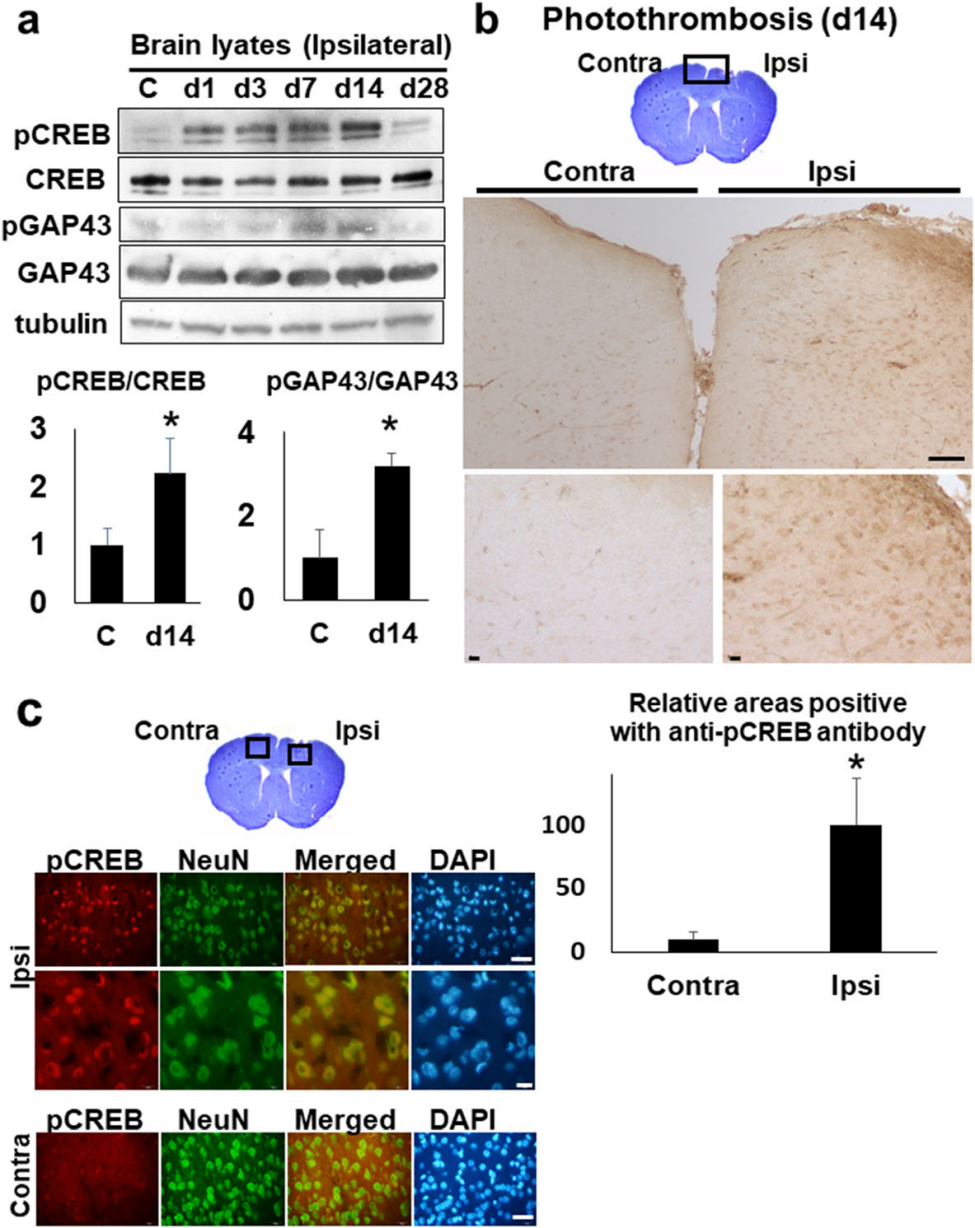
The activation of CREB and GAP in the stroke brain. **a** The western blot analyses with anti-CREB and -GAP43 antibody. The brain lysates (30 μg per each lane) of whole ipsi-lateral cortices at day 1, 3, 7, 14, and 28 were blotted with anti-CREB and anti-GAP43 antibody, respectively. C (control). The lower histogram shows the semi-quantification of band intensities of phosphorylated-CREB and -GAP43 relative to the total CREB and GAP43 protein (C vs. day 14), respectively (*p < 0.05, C vs. day14, Dunnett’s multiple comparison test). The value of control was made 1 for normalization. **b** The immunohistochemistry with ant-phosphorylated CREB (pCREB) antibody on the brain section, at day 14 post-stroke. The top picture shows the cresyl-violet stained bran section at day 14. Scale bar = 100 μm (upper panels), 10 μm (lower panels). The lower histogram shows the semi-quantification of DAB-stained areas positive with anti- pCREB antibody in ischemic brain sections (Contra- vs. Ipsi-lateral cortex) (*p < 0.05, Contra- vs. Ipsi-lateral, Student’s t-test). The value of ipsilateral brain was made 100 for normalization. Scale bar = 50 μm (upper panels), 10 μm (lower panels). **c** The immunofluorescence pictures of peri-ischemic regions on the Ipsi- and Contra-lateral cortex at day 14, with anti-phosphorylated CREB (pCREB) and anti-NeuN antibody, respectively. NeuN; Neuron-Specific Nuclear Protein. DAPI; 4′,6-diamidino-2-phenylindole. Scale bar = 50 μm (lower panels)

The transcription factor CREB (cAMP-response-element binding protein) enhances long-term synaptic plasticity and increases neuronal excitability [27], while the relatively neuron-specific growth-associated protein (GAP-43) is associated with presynaptic neuronal outgrowth and neuronal plasticity [28]. The phosphorylated-(Ser133) CREB and -(Ser41) GAP43 are active form implicated in neural plasticity, respectively. Then to investigate whether neural plasticity can occur in our model, we performed the western blot and immunohistochemistry using anti-CREB and anti-GAP43 antibody. The western blot analysis showed the induction of phosphorylated-(Ser133) CREB and -(Ser41) GAP43 at day 14 in the ipsilateral cortices (Fig. 7a). By immunohistochemistry, we found the up-regulation of phosphorylated CREB in the ipsilateral cortex (Fig. 7b) and the expression of phosphorylated CREB in the peri-ischemic neurons at day 14 post-stroke (Fig. 7c).

## Discussion

In the present study, we first conducted RNA-seq analyses to examine the temporal transcriptional response of the brain to the sterile neuroinflammation in the experimental stroke model during 28 days. Secondly, we focused on the temporal expression profiling of DAMPs-related genes using RNA-seq data. The gross morphological observation demonstrated the ischemic lesion apparently turned into a scar with debris clearance by day 28 post-stroke (Fig. 1a). The transcriptome analyses in Fig. 2c and d could support this observation, since the transcriptomic expression pattern at day 28 shows a similar tendency to that of control. The neuroinflammatory response-related genes were induced peaking at day 1 to 14 in the stroke brain (Supplementary Fig. S7), which are involved in microglial cell activation, astrocyte activation, and regulation of neuroinflammatory response. Previous studies showed the critical time limit for neural plasticity is considered to be around 30 days in rodent and 1–2 months in the human [29, 30], which suggests 28 days after stroke in the present study could be crucial for the neural reorganization. Interestingly, the increased number of microglial cells in the peri-ischemic regions continued even at day 28 post-stroke. Several genes (e.g., *s100a5*, *Cxcl5, C3, Mmp12*) were also induced even at day 28 (Fig. 5). Collectively, these findings suggest the inflammation, more or less, could continue beyond 28 days after stroke in this model.

The GO analysis of the biological process indicated that DEGs were genetically programmed to achieve the immune and inflammatory pathways (Fig. 3b, d). This is consistent with the concept that innate immune system is a rapid and coordinated defense response to eliminate the threat derived from not only infectious but also sterile insults [31, 32]. In accordance with this, neuroinflammatory response-related genes were induced, which are implicated in microglial cell activation, astrocyte activation, and regulation of neuroinflammatory response as described (Supplementary Fig. S7).

### Biphasic neuroinflammation in the post-ischemic brain

By hierarchical clustering and primary component analysis of RNA-seq data, we could classify the 6 temporal samples into 4 categories (I Baseline/Chronic, II Acute, III Sub-acute-#1, IV Sub-acute-#2 phase) (Fig. 2c, d). The transcriptomic pattern at day 14 (IV Sub-acute-#2) is characteristic, which is segregated from that of day 1 (I Acute) and day 3, 7 (III Sub-acute-#1). The human stroke period is clinically classified into three phases: the acute phase (first 48 h post-stroke), sub-acute phase (between 48 h to 6 weeks), and chronic phase (3or 6 months) based on the pathological characteristics [33]. We consider the sub-acute phase (III Sub-acute-#1 and IV Sub-acute-#2) in our study could correspond to the sub-acute phase in humans.

A subset of genes for inflammatory molecules, including cytokine (*Il1, Il6, Cxcl1, Cxcl2*), receptor (*Cxcr2*), *DAMPs* (*S100a8*), *Lcn2* (a master regulator of inflammation) [34], were biphasically induced peaking at the acute and sub-acute phases (Figs. 5 and 6, Supplementary Fig. S2, S3, S4 and S5). We assume this sub-acute phase could contribute to the reorganization of brain structure during the process to the chronic phase. In this phase, we observed the induction of genes for *TLRs* (*Tlr1, 2*, *7, 8*), *CLRs* (*Clec4d*, *4e*, *7a*), *Ccl5* (*Rante*), *Tgfβ*, *Ifnβ*, *IL10 receptors* (*Il10ra, Il10rb*), lysosomal enzymes (*Lyz1, 2*), and phagocytosis receptor (*Trem2*) and growth factors (*igf1, gdnf*) (Figs. 5 and 6). Among them, the induction of *Tgfβ*, *IL10 receptors, Lyz1*, and *Trem2* might be for the resolution of inflammation [5, 10, 11], while those of *igf1* and *gdnf* are considered as neuroprotective. The activation of microglia is defined as either classic (M1) or alternative (M2). M1 microglia secretes pro-inflammatory cytokines (e.g., TNFα, IL-1β) to exacerbate the neuronal injury, while M2 phenotype promotes anti-inflammatory responses possibly leading to the repair [35, 36]. This suggests the microglia in the sub-acute phase could be polarized to M2 phenotype. Genes for *DAMPs* (*S100a8*) and their receptor (*TLRs, CLRs*) are induced at this sub-acute phase (Figs. 5 and 6), raising the possibility that DAMPs might be implicated in the inflammatory response in the sub-acute phase, directly or indirectly.

TLR2, a representative toll-like receptor expressed on microglia of the ischemic brain, is critical for neuronal injury in the context of neuroinflammation [37], which was induced transcriptionally at the later phases in this study (Figs. 5 and 6). There is some controversy over the contribution of TLR2 to the outcome after ischemic stroke. One previous study with TLR2 knock out (KO) mice, subjected to focal brain ischemia, showed the lesion size reduced at day 3, however it increased at day 7 and 14 post-stroke [38]. This study showed the delayed exacerbation of the damaged area in TLR2 KO mice. In contrast, TLR2-deficient mice develop a decreased CNS injury compared to wild type mice in a model of focal cerebral ischemia [37]. The recent another study also reported TLR2-deficient mice to show the enhanced repair after focal brain ischemia with higher levels of Gap43 expression and caspases activity for neural plasticity [39]. Although the reason for this discrepancy is unknown, we speculate the moderate recurrent neuroinflammation at the sub-acute phase, if not over-activated, could contribute to the reorganization of ischemic brain.

### Phagocytosis and phosphorylated CREB at the sub-acute phase

The cellular debris on the ischemic lesion seemed cleared by day 28 post-stroke in Fig. 1a. The activated microglia (more rounded, hypertrophic, and amoeboid-like structure) appeared in peri-ischemic regions (Fig. 1c). We found the induction of phagocytosis-related genes at the sub-acute phase, including scavenger receptors (*Msr1*, *Trem-2-Tyrobp*, *Cd36*), complement receptors (*C3ar1*), and lysosomal enzymes (*Lyz1, 2*, *Ctsb, d, z*) (Fig. 5). Enhanced phagocytic activity leads to reduce the expression of pro-inflammatory mediators and increase the production of anti-inflammatory factors, thus resolving the neuroinflammation [10, 11]. In fact, we found the induction of genes for the regulatory molecules (e.g., *tgfβ, Il10ra, Il10rb*) as well as growth factors (e.g., *igf1, gdnf*) at this period (Fig. 5). In this context, microglia might be a major source of IGF-1 in the post-ischemic brain [40].

In addition, the number of down-regulated genes was the largest at day 14 (Fig. 2b) with significant enrichment in biological processes, including adhesion molecules, potassium ion transport, regulation membrane potential, chemical synaptic transmission, and neuron migration (Supplementary Fig. S5). The post-stroke neural plasticity includes two rules: (i) mechanisms of homeostatic plasticity, where hyper- and hypo-excitability regulate the appropriate synaptic inputs to neurons; and (ii) mechanisms of Hebbian plasticity, where synaptic strength is modified to favor synchronous networks inhibiting aberrant non-functional circuits [29]. We assumed the decreased expression of those genes at the sub-acute phase could contribute to the neural reorganization, which might regulate the excitability of synaptic inputs to neurons and aberrant non-functional circuits.

We found the induction of phosphorylated CREB and GAP at the sub-acute phase by western blot analysis (Fig. 7a). In addition, the immunohistochemistry showed phosphorylated CREB in peri-ischemic neurons (Fig. 7c). The recent study revealed that CREB overexpression enhances remapping of injured somatosensory and motor circuits, regulating the cortical circuit plasticity and functional recovery after stroke [27]. Collectively, the moderate recurrent inflammatory response at the sub-acute phase could contribute to the debris clearance as well as neural reorganization during the process to the chronic phase in the post-stroke brain.

Since most ischemic stroke occur in the territory of middle cerebral artery (MCA), the rodent MCA occlusion (MCAO) model has been extensively used in the field of stroke research. To compare the transcriptional response between MCAO and photothorombosis model, we examined the temporal expression profiling of DAMPs-related genes in the rat transient MCAO (tMCAO) model [41]. Using the available RNA-seq data of rat tMCAO model at day 1, 3, 7, 14, and 28 [41], we examined the temporal expression profiling of DAMPs-related genes in the same manner as our study (Supplementary Fig. S8). In the rat tMCAO model, DAMPs-related genes were induced, including DAMPs (*s100a8, s100a9*), receptors (*Tlr1, 2, 4, 6, 7,13, Clec2i, 4d, 4e, 5a, 7a),* signaling cascade (*Naip2, Naip5, Nlrp3, PYCARD, Irf7, Irf8*), cytokines (*Ccl2, 3, 4, 5, 6, 7, 8, 12, Cxcl1, 5, 9, 10, 13, 16, Ccr1, 2, 5, 7, 12, Cxcr2, 4*)*,* phagocytosis receptors (*Msr1*, *Cd36*, *Trem2-Tybrobp*), and complement (*C1qa, C1qb, C1qc, C3, C3ar1, C4b*). Interestingly, the expression peaks of these genes in the rat tMCAO model seemed relatively delayed in comparison with photothrombosis model (Fig. 5 and Supplementary Fig. S8), suggesting the inflammatory response in the rat tMCAO model could continue longer beyond 28 days after stroke. In addition, several genes were induced biphasically peaking at day 1–3 and day 14, including *S100a4*, *Clec4d, tnf, Ccl7,* and *Ccr5.*

There are several limitations in the present study. First and most importantly, this study is only descriptive and the contribution of DAMPs in the recurrent inflammation remains obscure. In addition, there are no direct evidence to know whether the recurrent inflammation could contribute to the debris clearance as well as neural reorganization.

## Conclusion

The temporal gene expression profiling revealed the biphasic neuroinflammation peaking at the acute and sub-acute phases in the post-stroke brain. Our findings raise the possibility the recurrent inflammatory response at the sub-acute phase could contribute to the debris clearance as well as neural reorganization during the process to the chronic phase.

## Supplementary information

**Additional file 1: Figure S1.** Top up-regulated genes and GO biological processes at day 3, 7 (d3, d7) post-stroke. **Figures S2-S4.** The results of qRT-PCR at each time point (control, day 1, 3, 7, 14, 28 post-stroke). **Figure S5.** Top down-regulated genes and GO biological processes at day 14 post-stroke. **Figure S6.** Semi-quantification of brain swelling and Iba1-stained cells. **Figure S7.** Temporal expression profiling for Neuroinflammation-related genes. **Figure S8.** DAMPs-related molecules in RNA-seq data of rat tMCAO model (BMC Genomics (2018) 19:655).

**Additional file 2: Table S1.** Primer list for real-time PCR.

**Additional file 3: Table S2.** DEGs at d1 post-stoke. **Table S3.** DEGs at d3 post-stoke. Supplementary **Table S4.** DEGs at d7 post-stoke. **Table S5.** DEGs at d14 post-stoke. **Table S6.** DEGs at d28 post-stoke.

## Data Availability

All primers for real-time PCR are shown in Supplementary Table S1. A detailed lists of the DEGs detected in each of the comparison groups are presented in Supplementary Tables S2, S3, S4, S5 and S6. The raw RNA-seq data have been deposited in NCBI’s Gene Expression Omnibus (GEO) and are accessible through GEO Series accession number GSE147060. The datasets used and/or analyzed during the current study are available from the corresponding author on reasonable request.
