# Supplementary material for: Temporal expression profiling of DAMPs-related genes revealed the biphasic post-ischemic inflammation in the experimental stroke model
Source: Mol Brain. 2020 Apr 7;13:57. doi: 10.1186/s13041-020-00598-1 (PMC7137489; doi:10.1186/s13041-020-00598-1)
Supplement: Supplementary file 1 — Additional file 1: Figure S1. Top up-regulated genes and GO biological processes at day 3, 7 (d3, d7) post-stroke. Figures S2-S4. The results of qRT-PCR at each time point (control, day 1, 3, 7, 14, 28 post-stroke). Figure S5. Top down-regulated genes and GO biological processes at day 14 post-stroke. Figure S6. Semi-quantification of brain swelling and Iba1-stained cells. Figure S7. Temporal expression profiling for Neuroinflammation-related genes. Figure S8. DAMPs-related molecules in RNA-seq data of rat tMCAO model (BMC Genomics (2018) 19:655). [file 13041_2020_598_MOESM1_ESM.pdf]

Supplementary data

Methods

RNA-sequencing library construction and sequencing

The total RNAs of 3 mice were pooled together at each time, including control (sham-operated mice). RNA samples were sent to the GENE WIZ (Saitama, Japan) for RNA-seq. Five hundred nanograms of total RNA per sample were used to construct sequencing libraries (pooled 3 mice at each time/ sample). Strand-specific RNA libraries were prepared using the NEBNext Ultra II Directional RNA Library Prep Kit for Illumina after poly(A) selection by the NEBNext poly(A) mRNA Isolation Module (New England Biolabs Inc.). Samples were barcoded using the recommended NEBNext Multiplex Oligos. Size range and quality of libraries were verified on the Agilent 2100 Bioanalyzer (Agilent Technologies). RNA-seq libraries were quantified by quantitative polymerase chain reaction using the KAPA library quantification kit (KAPA Biosystems). Each library was normalized to 2 nM and pooled in equimolar concentrations. Paired-end 150 sequencing was performed on an Illumina HiSeq4000 (Illumina, San Diego, CA). Libraries were pooled and sequenced using two lanes of one HiSeq4000 flow cell to an average depth of 33.6 million reads per sample.

Data analysis

Quality Control: In order to remove technical sequences, including adapters, polymerase chain reaction (PCR) primers, or fragments thereof, and quality of bases lower than 20, pass filter data of FASTQ format were processed by Trimmomatic (v0.30) to be high quality clean data.

Mapping: Firstly, reference genome sequences and gene model annotation files of relative species were downloaded from genome website (UCSC, NCBI, ENSEMBL). Secondly, Hisat2 (v2.0.1) was used to index reference genome sequence (mm10). Finally, clean data were aligned to reference genome via software Hisat2 (v2.0.1).

Expression analysis: In the beginning transcripts in FASTA format are converted from known gff annotation file and indexed properly. Then, with the file as a reference gene file, HTSeq (v0.6.1) estimated gene and isoform expression levels from the pair-end clean data.

Differential expression analysis: Differential expression analysis used the EdgeR. *p*-value of gene was set <0.05 to detect differential expressed ones. Data are shown in Supplementary Table S2-S6.

GO enrichment analysis: GO-TermFinder was used identifying Gene Ontology (GO) terms that annotate a list of enriched genes with a significant *p*-value less than 0.05. Genes with differential expression between groups were then included in gene ontology (GO) analysis to infer their functional roles and relationships. GO analysis for enriched GO biological processes in each set of differentially enriched genes identified by EdgeR was performed using DAVID (https://david.ncifcrf.gov).

Hierarchical clustering analysis: Hierarchical clustering analysis is to calculate and classify data according to similarity, so that samples or genes with similar expression patterns can be grouped together. This can assist to predict the function of unknown genes, and to predict whether they participate in the same metabolic process or cellular pathway. The FPKM (Fragments Per Kilobase of transcript per Million mapped reads) value of

different genes under different experimental conditions was taken as the expression level and used for hierarchical clustering. The regions of different colors in the dendrogram represent different clusters. Genes with similar expression patterns are within the same cluster and close to each other, and they may have similar functions or participate in the same biological processes.

PCA analysis: PCA (Principal Component Analysis) reduces data complexity helping to analyze sample relationship and the scales of the difference. The basic principle of PCA is to convert the original variables into a new set of independent variables (i.e., the principal components). All factors are ranked based on significance; minor factors and noise are eliminated, and thereby simplifies the data. Diagrams were made using two principal components as axes showing the clustering relationships between samples based on the distance between the various samples. Samples of close relationship tend to cluster together.

Supplementary Figure S1.

Top up-regulated genes and GO biological processes at day 3, 7 (d3, d7) post-stroke

a Top 20 Up-genes (d3) (p<0.05)

Symbol	Gene name	logFC	*p*-val
Tgm1	transglutaminase 1	7	2.28E-14
Mmp12	Matrix Metallopeptidase 12	11	8.05E-14
Arg1	Arginase 1	7	1.96E-13
Lcn2	Lipocalin-2	7	3.02E-13
Cd5l	CD5 Molecule Like	8	4.10E-13
Lgals3	Galectin 3	7	4.49E-13
Spp1	Secreted Phosphoprotein 1	6	2.23E-12
H19	gene for long noncoding RNA	6	3.22E-12
Lilr4b	Leukocyte Immunoglobulin Like Receptor B4	7	1.05E-11
Gpnmb	glycoprotein nonmetastatic melanoma protein B	5	1.95E-11
Ch25h	Cholesterol 25-hydroxylase	10	2.09E-10
Msr1	Macrophage Scavenger Receptor 1	5	3.26E-10
Hmox1	Homeobox A1	5	1.45E-09
Lyz2	lysozyme 2	4	1.41E-08
Timp1	Tissue inhibitor of metalloproteinase 1	4	6.22E-08
Plin2	Perilipin 2	4	1.07E-07
Lilrb4a	Leukocyte Immunoglobulin Like Receptor B4	4	1.71E-07
C3	Complement C3	4	2.46E-07
Cd300lf	CD300F immunoglobulin superfamily	6	2.54E-07
Tlr8	Toll Like Receptor 8	9	2.57E-07
Serpina3n	Serpin Family A Member 3	4	3.55E-07

b Top GO biological process (d3)

GO biological process	%	*p*-value
immune system process	11.6	9.9E-41
inflammatory response	10.7	1.3E-38
innate immune response	10.6	4.2E-33
chemotaxis	4.7	1.7E-20
mitotic nuclear division	6.4	6.7E-18
cell cycle	9.4	2.8E-16
immune response	6	7.1E-16
neutrophil chemotaxis	3.1	5.4E-15
cell division	6.9	7E-15
positive regulation of inflammatory response	2.9	1.3E-13
positive regulation of tumor necrosis factor production	2.4	6.8E-11
positive regulation of angiogenesis	3.1	6.5E-10
positive regulation of ERK1 and ERK2 cascade	3.9	1.1E-09
positive regulation of interferon-gamma production	2.1	1.2E-09
wound healing	2.7	2.1E-09

c Top 20 Up-genes (d7) (p<0.05)

Symbol	Gene name	logFC	*p*-val
Mmp12	Matrix Metallopeptidase 12	13	9.33E-19
Gpnmb	glycoprotein nonmetastatic melanoma protein B	7	7.08E-17
Cd5l	CD5 Molecule Like	9	1.91E-16
H19	gene for long noncoding RNA	8	2.55E-16
Spp1	Secreted Phosphoprotein 1	7	3.03E-15
Lgals3	Galectin 3	7	3.17E-14
Lyz2	lysozyme 2	6	1.18E-12
Clec7a	C-Type Lectin Domain Containing 7A	6	2.52E-12
Ch25h	Cholesterol 25-hydroxylase	11	6.39E-12
Lilr4b	Leukocyte Immunoglobulin Like Receptor B4	7	3.23E-11
Cst7	Cystatin F	6	5.05E-11
Avp	arginine vasopressin	6	9.05E-11
Mmp13	Matrix Metallopeptidase 13	10	2.99E-10
Itgax	Integrin Subunit Alpha X	6	5.57E-10
Atp6v 0d2	ATPase H+ Transporting V0 Subunit D2	10	6.41E-10
Msr1	Macrophage Scavenger Receptor 1	5	7.08E-10
Oxt	Oxytocin/Neurophysin	10	1.46E-09
Ccl3	Chemokine (C-C motif) ligand 3	7	1.75E-09
Gpr65	G Protein-Coupled Receptor 65	7	2.87E-09
Siglec1	Sialic Acid Binding Ig Like Lectin 1	5	5.76E-09

d Top GO biological process (d7)

GO biological process	%	*p*-value
immune system process	13.9	1.8E-60
innate immune response	11.3	5.6E-40
inflammatory response	8.9	9.7E-29
defense response to virus	5.6	1.4E-22
immune response	7	2.1E-22
chemotaxis	4.4	4.8E-19
response to virus	3.4	5.9E-16
neutrophil chemotaxis	2.6	1.6E-11
negative regulation of viral genome replication	1.9	1.9E-11
adaptive immune response	3.4	8.7E-11
positive regulation of cytokine secretion	1.8	8.1E-10
positive regulation of cell migration	3.8	2.5E-09
defense response to Gram-positive bacterium	2.6	3E-09
positive regulation of inflammatory response	2.2	3.1E-09
chemokine-mediated signaling pathway	2	4.1E-09

(a) (c) Table showing the top-upregulated genes (p<0.05) at day 3 and 7 post-stroke compared with control (sham), respectively. *logFC means log2 (fold changes).

(b) (d) Table showing the top-upregulated GO biological processes (p<0.05) enriched at day 3 and 7 post-stroke, which was identified by DAVID, respectively. * (%) means the ratio of up-regulated genes per total, which is involved in each biological process.

Supplementary Figure S2-S4.

The results of qRT-PCR at each time point (control, day 1, 3, 7, 14, 28 post-stroke).

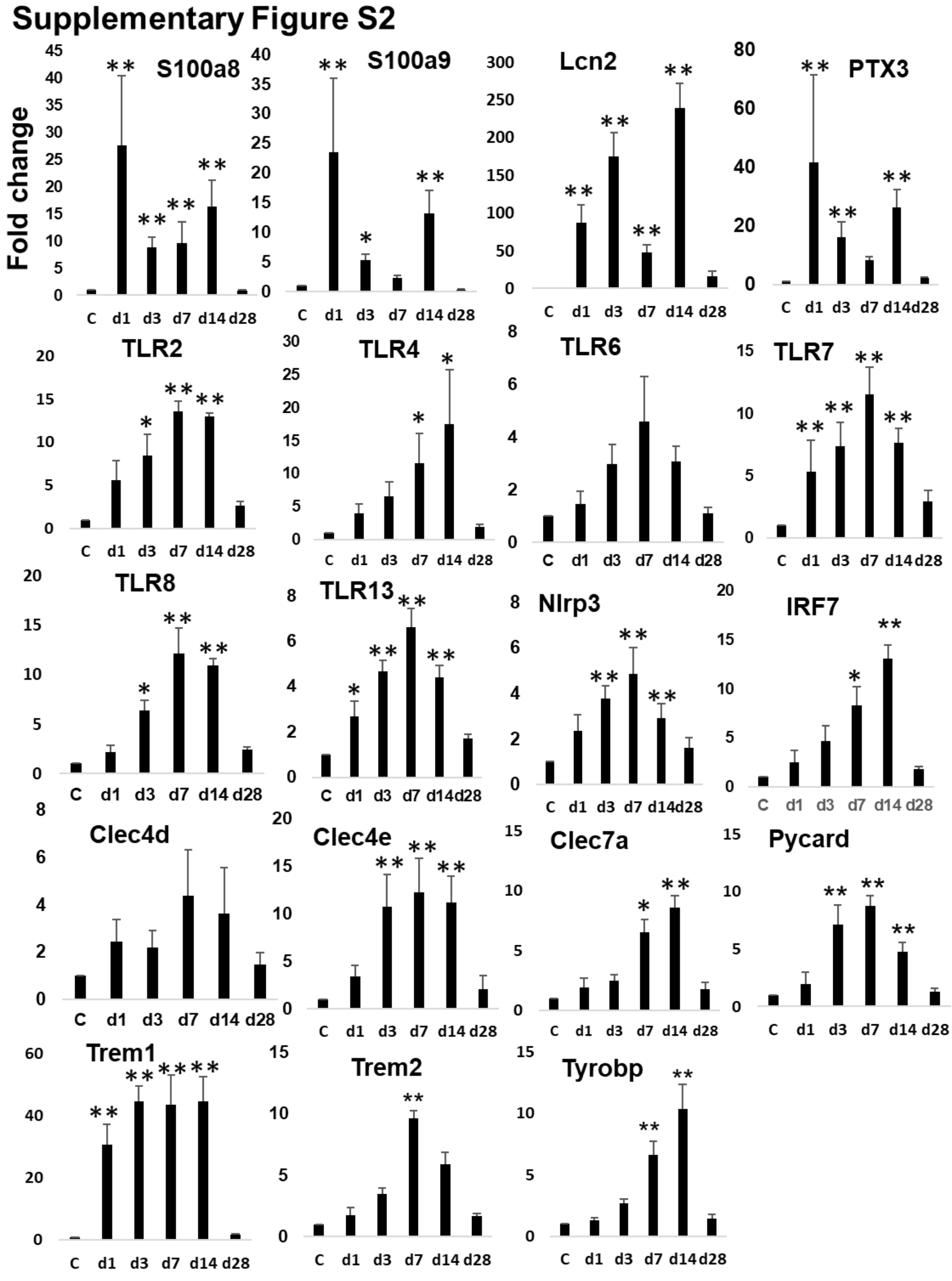

Histograms show the results of qRT-PCR at each time point (control, day 1, 3, 7, 14, 28 post-stroke). The results of cycle threshold values (Ct values) were calculated by the ΔΔCt method to obtain the fold differences. Five brains derived from each group (control and Photothrombosis) were used for real-time PCR analysis at each time point. Data are expressed as fold change vs sham-operated mice (control) (n=5/group). The bars represent the mean ± SEM (n = 5). The asterisks indicate a statistically significant difference from the control (sham-operated mice). (*$p<$ 0.05, **$p<0.01$, Dunnett's *multiple comparison test*).

Supplementary Figure S3

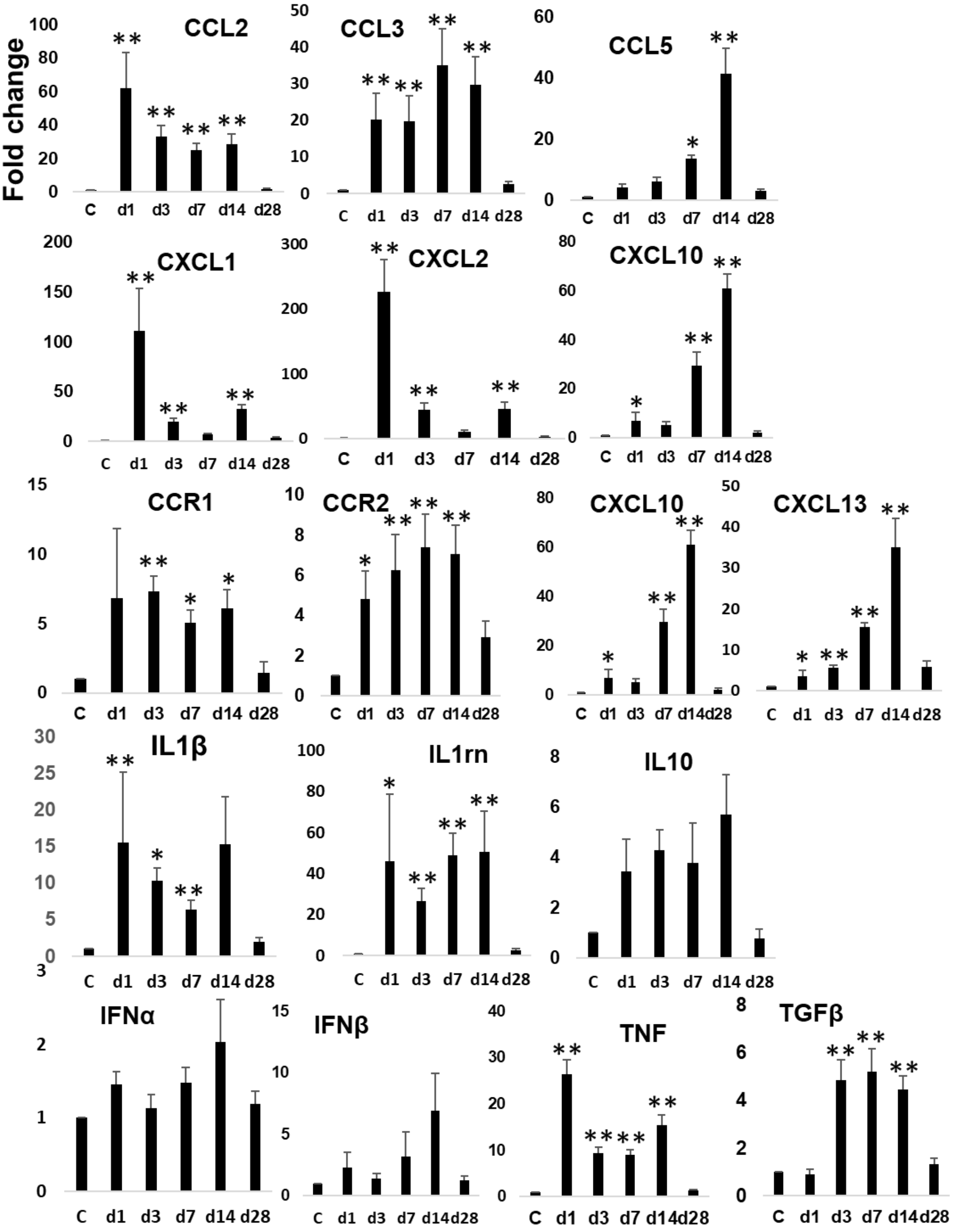

Histograms show the results of qRT-PCR at each time point (control, day 1, 3, 7, 14, 28 post-stroke). The results of cycle threshold values (Ct values) were calculated by the ΔΔCt method to obtain the fold differences. Five brains derived from each group (control and Photothrombosis) were used for real-time PCR analysis at each time point. Data are expressed as fold change vs sham-operated mice (control) (n=5/group). The bars represent the mean ± SEM (n = 5). The asterisks indicate a statistically significant difference from the control (sham-operated mice). (**p*< 0.05, ***p*<0.01, Dunnett's *multiple comparison test*).

Supplementary Figure S4

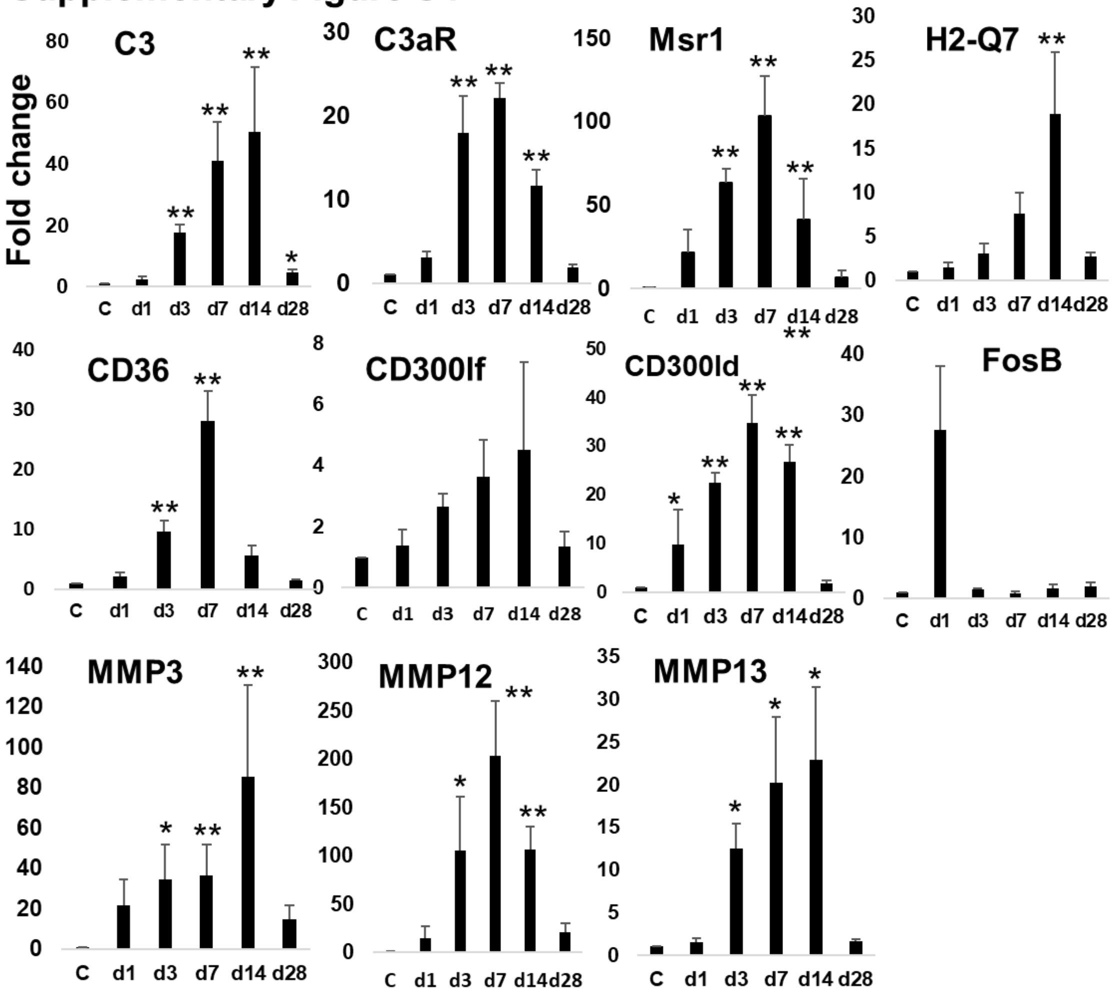

Histograms show the results of qRT-PCR at each time point (control, day 1, 3, 7, 14, 28 post-stroke). The results of cycle threshold values (Ct values) were calculated by the ΔΔCt method to obtain the fold differences. Five brains derived from each group (control and Photothrombosis) were used for real-time PCR analysis at each time point. Data are expressed as fold change vs sham-operated mice (control) (n=5/group). The bars represent the mean ± SEM (n = 5). The asterisks indicate a statistically significant difference from the control (sham-operated mice). (*p< 0.05, **p<0.01, Dunnett's *multiple comparison test*).

Supplementary Figure S5.

Top down-regulated genes and GO biological processes at day 14 post-stroke

a Top 20 Down-genes (d14) (p<0.05)

Symbol	Gene name	logFC	*p*-val
Dok6	a member of the DOK	-4.7	1.24E-08
Hs6st3	Heparan Sulfate 6-O-Sulfotransferase 3	-4.9	3.11E-08
Klf12	Kruppel Like Factor 12	-4.5	2.28E-07
Plag1	Pleomorphic adenoma gene 1	-4.6	1.27E-06
Kcnk9	Potassium channel subfamily K member 9	-3.7	1.56E-06
Gabrb2	GABA type A receptor beta2 subunit	-3.4	1.93E-06
Xkr4	XK Related 4	-3.5	2.89E-06
Dgkh	Diacylglycerol Kinase Eta	-3.4	4.75E-06
Dcc	Deleted in Colorectal Carcinoma	-3.5	1.13E-05
Zkscan16	ZNF483	-3.2	1.16E-05
Exph5	Exophilin 5	-3.3	1.17E-05
Capn11	Calpain 11	-3.9	1.19E-05
Zfp871	zinc finger protein 871	-3.2	1.51E-05
Lnpep	Leucyl-cystinyl aminopeptidase	-3.2	1.71E-05
Gpr165	G protein-coupled receptor 165	-3.3	2.32E-05
Kcna3	potassium voltage-gated channel subfamily A member 3	-3.4	2.33E-05
Fam205c	Family with sequence similarity 205 member C	-3.5	3.04E-05
Uprt	Uracil phosphoribosyltransferase homolog	-3.6	3.69E-05
Fam135b	Family With Sequence Similarity 135 Member B	-3.0	3.78E-05
Klhl11	Kelch Like Family Member 11	-3.0	3.83E-05

b Top GO biological process (d14)

	%	p value
homophilic cell adhesion via plasma membrane adhesion molecules	4.5	1.7E-06
potassium ion transport	3.7	0.00001
potassium ion transmembrane transport	3	0.000055
ion transport	6.3	0.00065
phospholipase C-activating G-protein coupled receptor signaling pathway	1.9	0.0041
regulation of membrane potential	2.2	0.0041
chemical synaptic transmission	2.6	0.011
neuron migration	2.2	0.011
adenylate cyclase-inhibiting G-protein coupled receptor signaling pathway	1.5	0.013
regulation of ion transmembrane transport	2.2	0.016
insulin receptor signaling pathway	1.5	0.018
positive regulation of cytosolic calcium ion concentration	2.2	0.023
cellular calcium ion homeostasis	1.9	0.023
exocytosis	1.9	0.027
phosphatidylinositol 3-kinase signaling	1.1	0.03
opioid receptor signaling pathway	0.7	0.032

(a) Table showing the down-upregulated genes ($p<0.05$) at day 14 post-stroke compared with control (sham). *logFC means log2 (fold changes).

(b) Table showing down-upregulated GO biological processes ($p<0.05$) enriched at day 14 post-stroke, which was identified by DAVID. *(%) means the ratio of down-regulated genes per total, which is involved in each biological process.

Supplementary Figure S6.

Semi-quantification of brain swelling and Iba1-stained cells

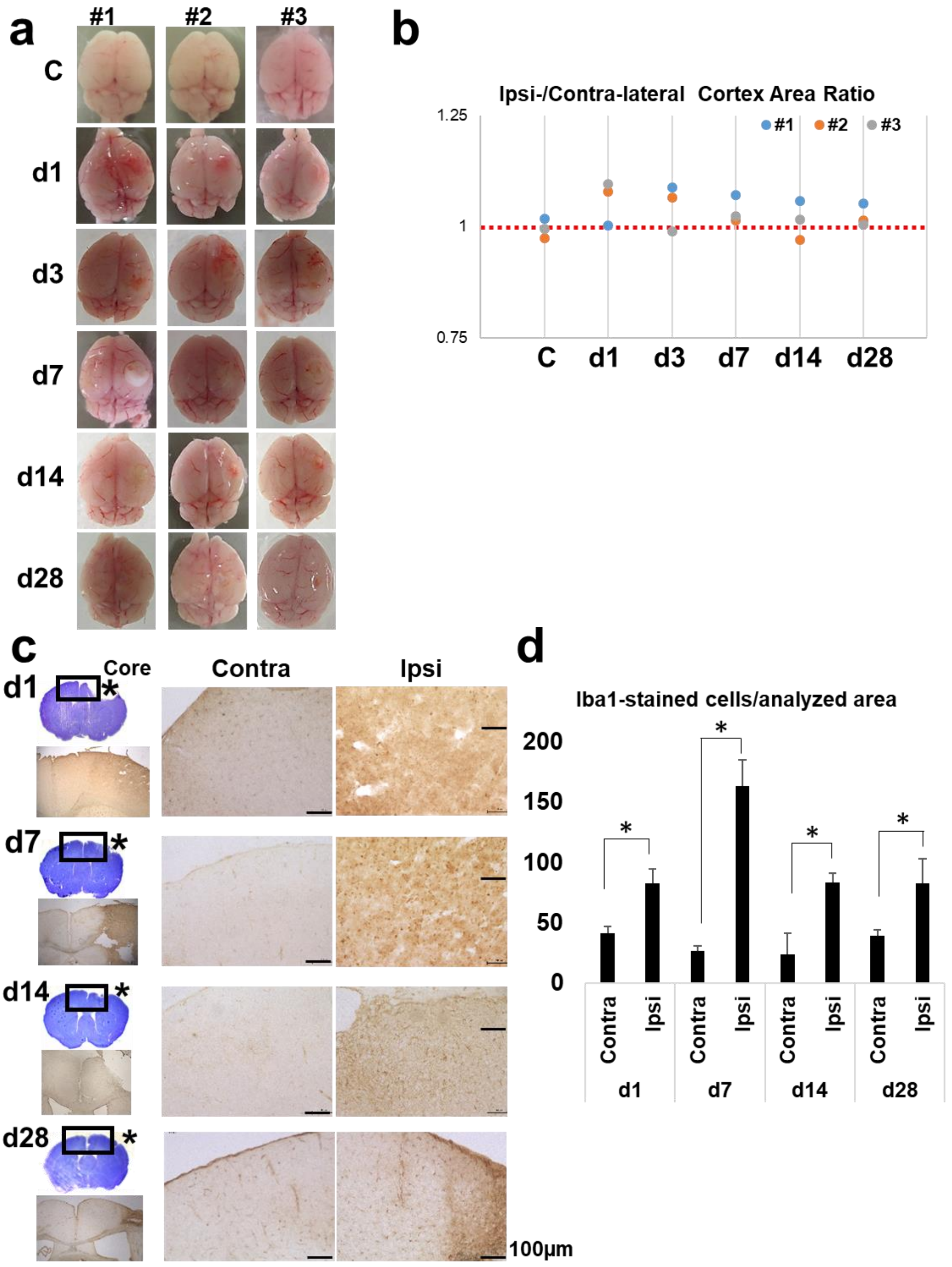

(a) Brain images of photothrombosis mouse model (#1, #2, #3) are shown at each time point. (b) The graph indicates the ratio of ipsi-/contra-lateral cortex area at each time point. (c) Representative section images (light microscopy, 2×, 10×) of Iba1-immunoreactivity in the peri-ischemic region and corresponding contra-lateral side, with Nissl stained images. (d) Quantifications are expressed as number of positive-stained cells/analyzed area. To semi-quantify the Iba1-immunoreactivity, the number of positive-stained cells/analyzed area were counted in at least five stroke brain sections (light microscopy 10×) on the ipsi- and contra-lateral side, respectively. Quantifications were expressed as the number of positive-stained cells/analyzed area at each time point. Values are means ± SD. Asterisk indicates a significant difference ($p < 0.05$, Student's t-test).

Supplementary Figure S7.

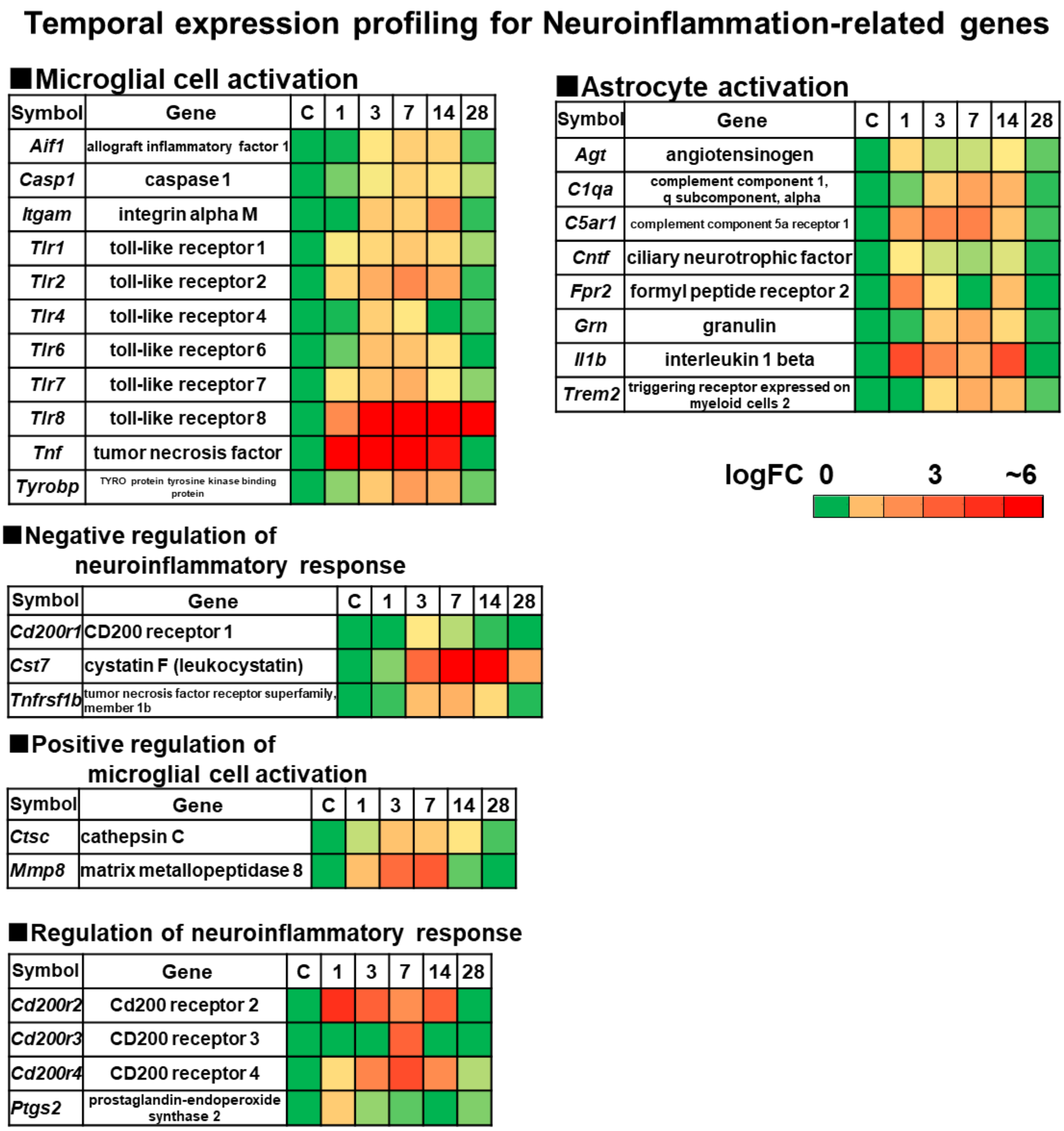

Heatmaps show the expression levels of neuroinflammatory response-related genes in RNA-seq data of photothrombosis model at each time point. The temporal profiles for genes that are transcriptionally induced (p<0.05) were categorized in each heatmap (gene name in row, logFC in column at each time point of control and day 1, 3, 7, 14, 28 post-stroke). The coloring range indicates the value of logFC. logFC means log2 (fold changes).

Supplementary Figure S8.

DAMPs-related molecules in RNA-seq data of rat tMCAO model (BMC Genomics (2018) 19:655**).**

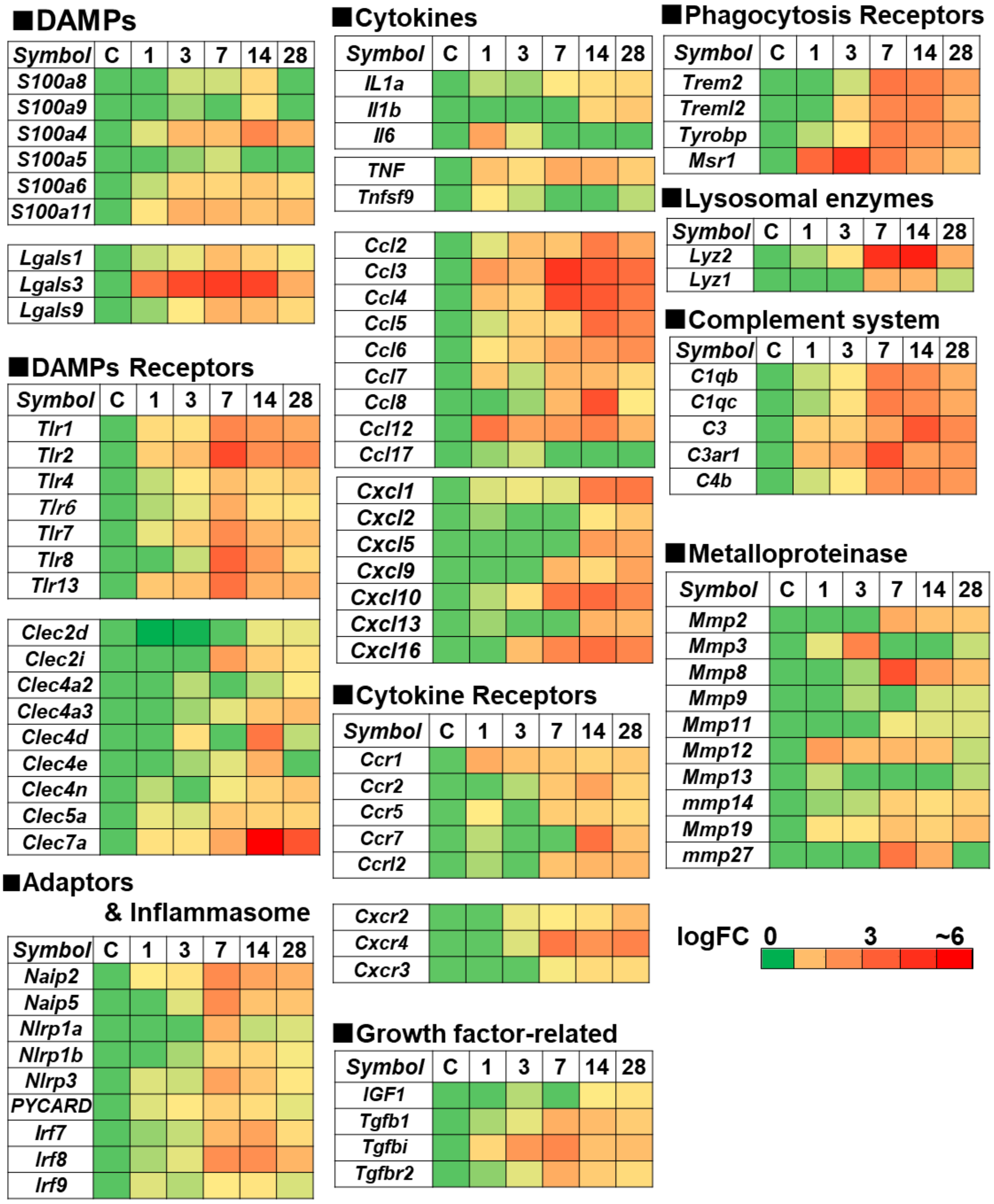

The temporal profiles for genes that are transcriptionally induced ($p<0.05$) were categorized in each heatmap (gene name in row, logFC in column at each time point of control and day 1, 3, 7, 14, 28 post-stroke) in rat tMCAO model. The coloring range indicates the value of logFC. logFC means log2 (fold changes).
