# Supplementary material for: Temporal expression profiling of DAMPs-related genes revealed the biphasic post-ischemic inflammation in the experimental stroke model
Source: Mol Brain. 2020 Apr 7;13:57. doi: 10.1186/s13041-020-00598-1 (PMC7137489; doi:10.1186/s13041-020-00598-1)
Supplement: Supplementary file 2 — Additional file 2: Table S1. Primer list for real-time PCR. [file 13041_2020_598_MOESM2_ESM.pdf]

Gene	Forward	Reverse	Reference
Lcn2	TTT CAC CCG CTT TGC CAA GT	GTC TCT GCG CAT CCC AGT CA	PLoS ONE 7(4): e34633
Fosb	CCTCCGCCGAGTCTCAGTA	CCTGGCATGTCATAAGGGTCA	PrimerBank ID 110350004c1
Msr1	TGGAGGAGAGAATCGAAAGCA	CTGGACTGACGAAATCAAGGAA	PrimerBank ID 164664519c1
Ccl2/MCP-1	GCATCCACGTGTTGGCTCA	CTCCAGCCTACTCATTGGGATCA	J Immunol January 1, 2011, 186 (1) 423-431
Ccl3/MIP-1	TGAAACCAGCAGCCTTTGCTC	AGGCATTCAGTTCCAGGTCAGTG	J Immunol January 1, 2011, 186 (1) 423-431
Ccl5	GCTGCTTTGCCTACCTCTCC	TCGAGTGACAAACACGACTGC	PrimerBank ID 7305461a1
CXCL1/KC	GCTTGAAGGTGTTGCCCTCAG	AAGCCTCGCGACCATTCTTG	J Immunol January 1, 2011, 186 (1) 423-431
CXCL2/MIP-2	GCGCTGTCAATGCCTGAAGA	TTTGACCGCCCTTGAGAGTG	J Immunol January 1, 2011, 186 (1) 423-431
CXCL10	CCAAGTGCTGCCGTCATTTTC	GGCTCGCAGGGATGATTTCAA	PrimerBank ID 10946576a1
C3	GACGCCACTATGTCCATCCT	CCAGCAGTTCCAGGTCCTTTG	The American Journal of Pathology 187(5)1186-1197, 2017
C3ar1	TCGATGCTGACACCAATTCAA	AGTCCCAATAGACAAGTGAGACC	PrimerBank ID 147904678c1
Cd5l	GGGGTTGACTGCAACGGAA	GGCCATCTACTAGACGCACA	PrimerBank ID 160358822c1
Cd300ld	ACATTGACCAAGCCCCAAAAA	AGCAGGGGTAACTCCACAAAG	PrimerBank ID 21703872a1
Cd300lf	CAGCAATCCAAGTACCCATTACA	CTGGTCTCTTTTACTGTGGTTGT	PrimerBank ID 25092716a1
Ch25h	ATGGGCTGCTACAACGGTTC	CCTTGTCCTTATGGTGTCCCAG	PrimerBank ID 6857768c1
H2-Q7	GAGCAGGCTGGTATTGCAGAG	CACCATAAGACCTGGGGTGA	PrimerBank ID 6754140a1
Mmp3	CAGACTTGTCCCGTTTCCAT	GGTGCTGACTGCATCAAAGA	Stroke Vasc Neurol. 2018 Sep; 3(3): 153–159
Mmp12	CCAAGCATCCCATCTGCTAT	GGTCAAAGACAGCTGCATCA	Stroke Vasc Neurol. 2018 Sep; 3(3): 153–159
Mmp13	TTTATTGTTGCTGCCCATGA	CTCTGGTGTTTTGGGATGCT	Stroke Vasc Neurol. 2018 Sep; 3(3): 153–159
Il1rn	TAGACATGGTGCCTATTGACCT	TCGTGACTATAAGGGGCTCTTC	PrimerBank ID 227116257c1
S100a8	AGTGTCCTCAGTTTGTGCAG	ACTCCTTGTGGCTGTCTTTG	J Leukoc Biol 82, 111-123.
S100a9	GTTGATCTTTGCCTGTCATGAG	AGCCATTCCCTTTAGACTTGG	J Leukoc Biol 82, 111-123.
Clec4e	AGTGCTCTCCTGGACGATAG	CCTGATGCCTCACTGTAGCAG	PrimerBank ID 9910162a1
Clec4d	GAA CAA ATT CTT GCC GTC CTG ACC	TCC ATC ACA AGG ACC ACT TTC TGA G	J Leukoc Biol. 2013 Sep; 94(3): 393–398.
Clec7a	GACTTCAGCACTCAAGACATCC	TTGTGTCGCCAAAATGCTAGG	PrimerBank ID 188035929c1
Lyz2	ATGGAATGGCTGGCTACTATGG	ACCAGTATCGGCTATTGATCTGA	PrimerBank ID 8393739a1
Cxcl13	GGC CAC GGT ATT CTG GAA GC	ACC GAC AAC AGT TGA AAT CAC TC	Neuroscience Bulletin doi: 10.1007/s12264-019-00377-6
Cxcr2	ATGCCCTCTATTCTGCCAGAT	GTGCTCCGGTTGTATAAGATGAC	PrimerBank ID 6753456a1
Ccr1	ACTGCTGTAAGAGCCTTTGGG	AGCACCAGAATCACTAGGACA	PrimerBank ID 157012006c1
Ccr2	TCCACGGCATACTATCAACATC	TCGTAGTCATACGGTGTGGTG	PrimerBank ID 160333416c1
TLR2	CAGCTGGAGAACTCTGACCC	CAAAGAGCCTGAAGTGGGAG	Neurological Sciences August 2013, Volume 34, Issue 8, pp 1339–1346
TLR4	CAACATCATCCAGGAAGGC	GAAGGCGATACAATTCCACC	Neurological Sciences August 2013, Volume 34, Issue 8, pp 1339–1346
TLR6	TGGATGTCTCACACAATCGG	GCAGCTTAGATGCAAGTGAGC	Neurological Sciences August 2013, Volume 34, Issue 8, pp 1339–1346
TLR7	TTCCTTCCGTAGGCTGAACC	GTAAGCTGGATGGCAGATCC	Neurological Sciences August 2013, Volume 34, Issue 8, pp 1339–1346
TLR8	TCTACTTGGCCTTGCAGAGG	ATGGCAGAGTCGTGACTTCC	Neurological Sciences August 2013, Volume 34, Issue 8, pp 1339–1346
TLR13	ATGGCACAAAACGGAGAAGAA	CTTTGTATACCCATGCCTCATCAG	Neurological Sciences August 2013, Volume 34, Issue 8, pp 1339–1346
IRF7	AGA GGG CGT TTT ATC TTG CG	TGG AGC CCA GCA TTT TCT CT	J Biol Chem. 2016 Jun 17;291(25):13206-15
Nlrp3	ATCAACAGGCGAGACCTCTG	GTCCTCCTGGCATACCATAGA	J Immunol 2017; 199:1660-1671;
Pycard	GACAGTGCAACTGCGAGAAG	CGACTCCAGATAGTAGCTGACAA	J Immunol 2017; 199:1660-1671;
Cd36	TTGAAAAGTCTCGGACATTGAG	TCAGATCCGAACACAGCGTA	https://doi.org/10.1073/pnas.1505675112
Trem1	GACTGCTGTGCGTGTTCTTTG	GCCAAGCCTTCTGGCTGTT	PrimerBank ID 10946760a1
Trem2	GGAACCGTCACCATCACTCT	GGAGGTGCTGTGTTCCACTT	Primer3
TYROBP(DAP12)	GAGTGACACTTTCCCAAGATGC	CCTTGACCTCGGGAGACCA	PrimerBank ID 6755915a1
IL-10	AGTGGAGCAGGTGAAGAGTG	TTCGGAGAGAGGTACAAACG	DOI: 10.1128/MCB.00143-15 Published 17 August 2015
TGF-β1	GCTACCATGCCAACTTCTGT	CGTAGTAGACGATGGGCAGT	DOI: 10.1128/MCB.00143-15 Published 17 August 2015
IL-1β	CCCAACTGGTACATCAGCAC	TCTGCTCATTCACGAAAAGG	DOI: 10.1128/MCB.00143-15 Published 17 August 2015
TNFα	CCCACTCTGACCCCTTTACT	TTTGAGTCCTTGATGGTGGT	DOI: 10.1128/MCB.00143-15 Published 17 August 2015
IFN-α	GGACTTTGGATTCCCGCAGGAGAAG	GCTGCATCAGACAGCCTTGCAGGTC	Liu et al. 10.1073/pnas.0901216106
IFN-β	AACCTCACCTACAGGGCGGACTTCA	TCCCACGTCAATCTTTCCTCTTGCTTT	Liu et al. 10.1073/pnas.0901216106
